# Isolation and long‐term culture of primary mouse cholangiocytes that retain biophysical properties and distinct Cl‐conductances: An initial study

**DOI:** 10.14814/phy2.70732

**Published:** 2026-01-20

**Authors:** Qin Li, Youxue Wang, Kristy Boggs, Charles Kresge, Kari Nejak‐Bowen

**Affiliations:** ^1^ Department of Pediatrics University of Pittsburgh Medical Center (UPMC) Pittsburgh Pennsylvania USA; ^2^ Department of Pediatrics University of Texas Southwestern Medical Center Dallas Texas USA; ^3^ Organ Pathobiology and Therapeutics Institute, University of Pittsburgh School of Medicine Pittsburgh Pennsylvania USA; ^4^ Department of Pharmacology and Chemical Biology University of Pittsburgh School of Medicine, Pittsburgh Pittsburgh Pennsylvania USA; ^5^ Pittsburgh Liver Research Center, University of Pittsburgh and University of Pittsburgh Medical Center Pittsburgh Pennsylvania USA

**Keywords:** bile duct epithelial cells, chloride currents, cystic fibrosis transmembrane conductance regulator, patch clamp

## Abstract

Cultures of primary mouse bile duct epithelial cells are a valuable tool to study cholangiocyte secretion and bile formation. However, freshly isolated cells have a limited ability to expand in culture. Here we report a novel isolation and culture technique for normal mouse cholangiocytes (NMC) that enables long‐term growth without compromising function. Mouse cholangiocytes were isolated and cultured in conditioned medium (CM) that was subsequently supplemented with ROCK inhibitor Y‐27632. Expression of cholangiocyte markers was assessed by qPCR, immunofluorescence, and Western blotting. Patch clamp techniques were used to measure cAMP‐activated Cl‐ current, Ca2+−activated Cl‐ current, and volume‐stimulated Cl‐ current. We obtained NMC cultures that were polarized and maintained a cholangiocyte phenotype for over 50 passages. Functional studies show that ion channel activity is maintained in NMC regardless of the number of passages and despite removal of CM. NMC also perform other physiological functions such as ATP release and intracellular Ca2+ changes in response to stimulation with bile acids. Thus, our isolation procedure produces viable NMC that maintain biophysical properties in long‐term culture. We also demonstrate the utility of NMC in studies investigating the cellular mechanisms responsible for cholangiocyte secretion and bile formation.

## Graphical abstract

Mouse cholangiocytes are isolated from bile ducts and cultured in conditioned medium containing ROCK inhibitor. Cells maintain expression of biliary markers & ion channel activity for >50 passages.

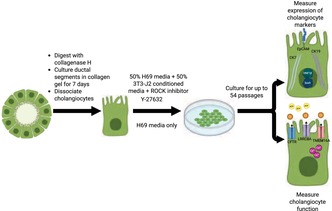

## 1 INTRODUCTION

Cholangiocytes are epithelial cells that line the intrahepatic bile ducts. The major physiologic function of cholangiocytes is bile formation and transport. Bile formation is a complex process regulated by hormones, peptides, nucleotides, neurotransmitters, and biliary constituents. Cholangiocytes modify primary (canalicular) bile secreted by hepatocytes through a series of secretory and re‐absorptive processes. This is achieved by the coordinated vectorial transport of selected ions, solutes, and water across the cholangiocyte apical and basolateral plasma membranes via numerous channels, transporters, and exchangers (Banales et al., 2019; Jalan‐Sakrikar et al., 2025; Tabibian et al., 2013).

While primary fresh isolated mouse cholangiocytes accurately represent the phenotype of cells in vivo, daily isolation and the need for immediate use is a barrier for widespread adoption. Freshly isolated cells have a limited ability to expand in sufficient numbers in culture. Because of this, much of the research in the mouse cholangiocyte field has been carried out with commercially available cell lines of malignant origin. (Mano et al., 1998; Sirica et al., 1990; Ueno et al., 2003) Recently, cholangiocyte‐like cells from induced pluripotent stem cells and cholangiocyte‐derived organoids have provided new sources of biliary cells for study. (Chen et al., 2022; Florentino et al., 2022; Luce & Dubart‐Kupperschmitt, 2020; Sampaziotis et al., 2021) However, for the most part these protocols prioritize morphological characterization and lack functional assessment. Methods that achieve long‐term culture and cloning of primary mouse cholangiocytes that maintain multipotent differentiation capacity and Cl‐ channel function would therefore be advantageous.

A recent study showed that conditional reprogramming of cells with a Rho‐associated protein kinase (ROCK) inhibitor extended the life span of primary human bronchial epithelial cells (Peters‐Hall et al., 2018) The technique conditionally maintains epithelial cells in a stem cell‐like state that enables long‐term growth. In the present report, we describe the adaptation of these methods to the long‐term culture of normal mouse cholangiocytes (NMC) from mouse liver. Our isolation procedure reproducibly yields viable cholangiocytes that can be successfully passaged and expanded to create stable cell lines. We then tested our method through functional studies that measure retention of biophysical properties and distinct chloride (Cl‐) conductance, key biological processes relevant to cholangiocytes. We have explored three different Cl‐ channels in NMC cells and show that these cells retain normal physiological function in both early and late passages. The techniques described herein thus provide a method of maintaining long term cultures of cholangiocytes and should prove to be a valuable tool in the field of biliary biology.

## 2 METHODS

### 2.1 Animals

A total of 5 wild‐type (WT) male mice and 2 CFTR knockout (KO) male mice were used for the cell isolation methods described in detail below. All animal studies were performed following the guidelines of the Institutional Animal Use and Care Committee at the University of Pittsburgh School of Medicine and the National Institutes of Health (Protocol number: IS00018809). All mice were in a C57Bl/6 background (Jackson Laboratories) and maintained in ventilated cages under 12 h light/dark cycles with access to enrichment and water. WT mice consumed mouse chow (5P76 Irradiated PicoLab IsoPro RMH 3000; LabDiet, St. Louis, MO) ad libitum.

CFTR KO mice (CFTRtm^1UNC^) (Snouwaert et al., 1992) have high rates of spontaneous death (90%) due to intestinal obstruction around the time of weaning at 3 weeks of age. To significantly reduce spontaneous death (from 90% down to 20% or less), upon weaning at 3 weeks of age, KO mice were placed on a complete liquid diet of Peptamen (HCPCS code B4153; Nestle Clinical Nutrition, Deerfield, IL) ad libitum. Each mouse was given 20 mL of Peptamen on a daily basis, and fresh Paptamen was supplied every day.

### 2.2 Media and solutions

*Tissue wash solution*: DMEM supplemented with penicillin (100 units/mL), streptomycin (100 units/mL), and amphotericin B (25 ng/mL) *Tissue digestion solution*: Tissue wash solution with collagenase H (1 mg/mL; from *Clostridium histolyticum*, catalog no. 11074032001, Roche, Basel, Switzerland), and hyaluronidase (0.025 mg/mL, from bovine testes, H3506, Sigma‐Aldrich, Sigma‐Aldrich, St. Louis, MO). *Cell wash solution*: PBS with 0.5% of bovine serum albumin (BSA; A4737, Sigma‐Aldrich) and 2 mM of EDTA.

*H69 culture medium*: DMEM low glucose (50%), DMEM/F12 (50%), with 10% fetal bovine serum (FBS; MT35010CV, Fisher Scientific, Waltham, MA), Penicillin (100 units/mL), and streptomycin (100 units/mL), Adenine HCL (30 μg/mL; AAA1490618, Fisher Scientific), Insulin (5 μg/mL; I5500; Sigma‐Aldrich), (±) epinephrine (1 μg/mL; E4642, Sigma‐Aldrich), Epidermal growth factor (human) (10 ng/mL; CB‐40052, Fisher Scientific), T3 + T (3,3′5‐triiodo‐L‐thyronine + Apo‐transferrin) (2.27 ng/mL + 8.34 μg/mL; AAJ63312ME and 3188AT100MG, Fisher Scientific), Hydrocortisone (13 μg/mL; AC352450010, Fisher Scientific).

*3T3‐J2 culture medium*: DMEM with 10% fetal calf serum (FCS), Penicillin (100 units/mL), and streptomycin (100 units/mL).

*Conditioned medium*: 3T3‐J2 cells (Kerafast, Boston, MA) (Rheinwald & Green, 1975) were cultured to 70%–90% confluence with 3T3‐J2 culture medium. They were then treated with mitomycin (5 μg/mL; MP210049802, Fisher Scientific) for 3 h. After the cells were washed with PBS 3 times and with culture medium once, they were cultured with H69 medium for 3 days. Then the culture medium (H69) was collected, filtered through a 0.22 μm filter, and stored at –80°C until being used as conditioned medium.

### 2.3 Reagents

The Forskolin (catalog no. F‐9929) was obtained from LC Laboratories (LC Laboratories, Woburn, MA), 4‐[(2‐butyl‐6,7‐dichloro‐2‐cyclopentyl −2,3‐dihydro‐1‐oxo‐1H‐inden‐5‐yl) oxy] butanoic acid (DCPIB; catalog no. 1450) was from Tocris, and IBMX (catalog no. I5879), 172 inhibitor (catalog no. C2992), apyrase (A6535), and norUDCA (catalog no. SML3481) were from Sigma‐Aldrich (St. Louis, MO).

### 2.4 Cell isolation

#### 2.4.1 Bile duct cell isolation

Liver lobes were resected 2–5 mm distal to the entry sites of hepatic ducts from 3‐month‐old male mice weighing approximately 28 grams. The liver lobe capsules were removed, and multiple tears were made in the liver lobes with forceps. The liver tissue was then digested with collagenase H (1 mg/mL) and hyaluronidase (0.025 mg/mL) at room temperature and monitored under a microscope. Parenchymal hepatic tissue and connective tissue were removed with enzyme digestion in combination with physical removal and separation with needles. The duct segments were saved and cultured in H69 medium at 37°C with 5% CO_2_.

After overnight culture, the segments of duct with sealed ends and clear lumen were sliced open and cut into small pieces. The diced ductal tissue was cultured in 0.65 mg/mL collagen gel prepared with PureCol EZgel (bovine neutralized type I collagen solution, catalog #5074; Advanced BioMatrix, San Diego, CA) and H69 culture medium. After 1 week of culture, the collagen gel was digested with collagenase (1 mg/mL) and dispase (0.2 unit/mL; catalog no. D4818, Sigma‐Aldrich) for 1 h to dissociate the cholangiocytes. Both the suspended cells and cells that were attached to the bottom of the culture dish were cultured for 2 more days before immune affinity separation.

#### 2.4.2 Bile duct cell immune separation

The cells were trypsinized and treated with DNase (0.5 mM, catalog no. 10104159001, Roche) in PBS for 35 min. After the cells were washed with PBS, they were treated with fresh DNase (1 mM) for an additional 15 min. The cells were then pushed through a 100 μm Falcon cell strainer (catalog no. 352360, Corning Life Sciences, Tewksbury, MA). After centrifugation, the cells were re‐suspended in 1 mL of PBS with 0.5% of bovine serum albumin (BSA) and 2 mM of EDTA and were then incubated with 20 μL of Purified Rat Anti‐Mouse CD326/Epithelial Cell Adhesion Molecule (EpCAM) antibody (catalog no. 552370, BD Biosciences, San Jose, CA) at 4°C on a shaker for 1 h. After the cells were washed twice, they were resuspended in 240 μL of PBS with 0.5% BSA and 2 mM EDTA and incubated with 60 μL of Anti‐Rat Microbeads (catalog no. 130‐048‐502, Macs Miltenyi Biotec, Auburn, CA) at 4°C for 10 min. After the cells were washed twice, they were resuspended in 500 μL of PBS with 0.5% BSA and 2 mM EDTA and applied on an equilibrium column in the magnetic field. After the column was washed 3 times, the magnetic field was removed, and the cells were eluted from the column. The cells were then cultured in H69 medium at a density of one to several hundred, depending on the yield after isolation and magnetic purification.

#### 2.4.3 Long‐term expansion under conditional reprogramming conditions

Two days after separation, cells were cultured in H69 medium (66.66%) with 3T3‐J2 cell conditioned medium (33.3 3%) and ROCK inhibitor Y‐27632 (5 μM, catalog no. ALX‐270‐333, Enzo, Farmingdale, NJ) for 3 weeks, and then with 50% H69 medium, 50% conditioned medium, and 10 μM Y‐27632. The conditioned medium was prepared by treating 70%–90% confluent 3T3‐J2 (Kerafast, Boston, MA) with mitomycin C (5 μg/mL) for 3 h, culturing them with H69 medium for 3 additional days, and collecting the culture medium. The conditioned medium was filtered through a 0.22 μm filter and stored at −80°C until use. Long‐term cultures were created from *n* = 2 WT and 1 CFTR KO mice.

#### 2.4.4 Cell counting

Cells harvested 1, 2, 3, and 4 weeks after plating were diluted with 0.2% trypan blue and counted using a hemocytometer. Triplicate wells were analyzed for each time point.

### 2.5 Measurement of cystic fibrosis transmembrane conductance regulator (CFTR) currents

Membrane CFTR currents were measured by whole cell patch clamp techniques. Cells on a cover slip were mounted in a chamber (volume ~ 400 μL) and perfused at 2 ~ 4 mL/min with an extracellular solution containing 150 mM *N*‐methyl‐d‐glucamine‐Cl (catalog no. A755677, Sigma‐Aldrich), 1 mM MgCl_2_, 1 mM CaCl_2_ and 10 mM Hepes‐Tris at pH 7.4. The osmolarity of the standard solution was 300 ± 10 as measured by a vapor pressure osmometer (Advanced Micro Osmometer, MA, USA). The internal pipette solution was composed of 150 mM *N*‐methyl‐d‐glucamine‐Cl, 1 mM MgCl_2_, 1 mM EGTA, 0.5 mM ATP and 10 mM HEPES at pH 7.3. The [Ca2+]_i_ was buffered in the presence of 10 mM BAPTA (catalog no. AAA1319014, Fisher Scientific) to 1 nM or to other intracellular concentrations as indicated. Patch pipettes were pulled from Corning 7052 glass and had a resistance of 5–7 MΩ. Recordings were made with an Axopatch 200B amplifier (Axon Instruments, Foster City, CA), and were digitized (1 kHz) for storage on a computer and analyzed using pCLAMP version 11.0.3 programs (Axon Instruments, Burlingame, CA) as previously described (Feranchak et al., 2001) The voltage protocol applied a holding potential −40 mV with a ramp from −100 mV to +100 mv over 450 ms at 2 s intervals (for real‐time tracings). The step protocol used a holding potential −40 mV, with steps from −100 mV to +100 mV over 450 ms in 20 mV increments. Pipette voltages (Vp) correspond to the membrane potential, and upward deflections of the current trace indicate outward membrane current. Results are compared with control studies measured on the same day to minimize any effects of day‐to‐day variability and reported as current density (pA/pF) to normalize for differences in cell size. Capacitance and access resistance were monitored continuously.

### 2.6 Measurement of transmembrane member 16A protein (TMEM16A) Cl‐ currents

Cells on a coverslip were mounted in the chamber, and whole cell currents were measured during basal and perfused conditions with a standard extracellular solution containing (in mM) 140 NaCl, 4 KCl, 1 CaCl2, 2 MgCl2, 1 KH2PO4, 10 glucose, and 10 HEPES/NaOH (pH 7.40). The standard intracellular (pipette) solution for whole cell recordings contained 130 mM KCl, 10 mM NaCl, 2 mM MgCl2, 10 mM HEPES/KOH, 0.5 mM CaCl2, and 1 mM EGTA (pH 7.3), corresponding to a free Ca2+ concentration of 100 nM. Patch pipettes, which were pulled from Corning 7052 glass, had a resistance of 4–6 M. Recordings were made with an Axopatch 200B amplifier (Axon Instruments, Foster City, CA), digitized (2 kHz), and analyzed using pCLAMP version 11.0.3 (Axon Instruments, Burlingame, CA), as previously described. (Dutta et al., 2011) Two voltage protocols were utilized: (1) holding potential of −40 mV with 450 ramps from −100 mV to +100 mV at 2‐s intervals and (2) holding potential of −40 mV with 450‐ms steps from −100 to +100 mV in 20 mV increments. Current–voltage relations were generated from the “step” protocol. Results are compared with control studies measured on the same day to minimize effects of day‐to‐day variability and reported as current density (pA/pF) to normalize for differences in cell size (Feranchak et al., 2001).

### 2.7 Measurement of volume‐activated Cl‐ currents

Coverslips with NMC cells were transferred to the recording chamber and perfused with a standard external solution with the following composition: 145 mM NaCl, 1 mM CaCl2, 1 mM MgCl2, 10 mM HEPES, and 10 mM D‐glucose, pH 7.4 (NaOH). Osmolarity was adjusted with mannitol to 310 ± 5 mOsm using the Advanced Micro Osmometer (Model 3300, Advanced Instruments Inc.). For measuring swelling‐activated currents and volume changes, an isotonic solution containing 85 mM NaCl, 1 mM CaCl2, 1 mM MgCl2, 10 mM HEPES, 120 mM D‐mannitol, and 10 mM D‐glucose, pH 7.4, with NaOH (310 ± 5 mOsm) was used and cell swelling was induced by omitting mannitol from the solution to reach osmolarity of 190 mOsm for most volume‐activated studies (Shcheynikov et al., 2022) Patch‐clamp experiments were performed in the standard whole‐cell configuration at room temperature (22°C–25°C) using an Axopatch 200B amplifier (Axon Instruments). Patch pipettes had resistances between 4 and 6 mΩ after filling with the standard intracellular solution that contained the following: 90 mM cesium (Cs)‐chloride, 50 mM Cs‐aspartate, 1 mM Mg‐ATP (catalog no. A9187, Sigma‐Aldrich), 10 mM HEPES, and 2 mM EGTA, pH 7.3 (cesium hydroxide). Digidata −1440A and pClamp 13 software (Molecular Devices) were used for data acquisition and analysis. The current was recorded by 450‐ms rapid alteration of membrane potential from −100 to +100 mV every 2 s from a holding potential of −40 mV. The current recorded at +100 mV was used to calculate the maximum current density (pA/pF). To observe the voltage and time dependency of the current profile, step pulses were applied from a holding potential of −40 mV to test potentials of −100 to +100 mV in +20 mV increments. The current was filtered at 1 kHz and sampled at 10 kHz. Results are compared with control studies measured on the same day to minimize any effects of day‐to‐day variability and reported as current density (pA/pF) to normalize for differences in cell size. Data are presented as mean ± SD. Origin 2018 (OriginLab) was used for data analysis and display.

### 2.8 Ca2+ imaging

Cells were cultured for 24 h on 10 mm glass coverslips and then loaded with 2.5 μg/mL of fura‐2 am (catalog no. 1051B; TEF Laboratories, Austin, TX, USA) in isotonic extracellular buffer containing: 140 mM NaCl, 4 mM KCl, 2 mM CaCl_2_, 1 mM MgCl_2_, 1 mM KH_2_PO_4_, 10 mM glucose, 10 mM HEPES (pH 7.4) supplemented with 0.01% pluronic F127 for 30 min at 37°C. The coverslip was placed in the perfusion chamber on the stage of an inverted fluorescent microscope (Nikon TE2000). Changes of [Ca2+]_i_ were measured at excitation wavelength of 340 nm for calcium‐bound fura‐2 am and 380 nm for calcium‐free fura‐2 am, emission wavelength of 510 nm. Experiments were performed at room temperature. Ca2+ studies were conducted on 20–30 cells per field and repeated in three technical replicates.

### 2.9 Measurement of ATP release

Cellular ATP release was studied using the luciferin‐luciferase (L‐L) assay as previously described (Feranchak et al., 2000; Taylor et al., 1998) Cells on 35 mm tissue‐culture‐treated dishes (Falcon, Becton Dickinson Labware, Franklin Lakes, NJ) were washed with PBS (600 μL × 2), 600 μL Optimem (Gibco) containing L‐L (ATP Assay Kit, #FLAA, Sigma‐Aldrich) added, and then placed into a modified Turner TD 20/20 Luminometer. After a 10‐min equilibration period, a basal reading was obtained, and then an equal volume of isotonic buffer (200 μL) was added (to account for ATP release due to mechanical stimulation), followed by the addition of hypotonic buffer. Readings were performed as cumulative bioluminescence over a 15 second interval and quantified as arbitrary light units (ALU's). Standard calibration curves were performed with known amounts of ATP added to the L‐L Optimem reagent during cell‐free conditions. All luminescence values are reported as relative change from basal luminescence per total protein level in the sample (measured in μg per ml) to control for any potential differences in luciferase activity or confluency between samples, respectively (Woo et al., 2010).

### 2.10 Immunofluorescence

Localization of CFTR, TMEM16A, CK19, and LRRC8A was performed in confluent NMC monolayers. NMC on rat‐tail collagen type I coated tissue culture chamber slides (BD BioCoat #354630; Corning Life Sciences) were fixed in 25% acetic acid/75% ethanol (v/v) for 10 min or 4% paraformaldehyde permeabilized with 1% Triton‐X 100 for 10 min, incubated with 5% normal donkey serum (catalog no. 017–000‐001; Jackson Immunoresearch Laboratories, West Grove, PA), and then incubated overnight at 4°C with either rabbit anti‐CFTR antibody (ACC‐034, Alomone Labs, Jerusalem, Israel 1:200), anti‐TMEM16A (ABN1669, Chemicon, 1:200), anti‐CK19 (10712‐1‐AP, Proteintech, 1:200), or anti‐LRRC8A (ACC‐001, Alomone Labs, 1:200), followed by 488 conjugated donkey anti‐rabbit (catalog no. 711‐545‐152, Jackson Immunoresearch Laboratories, 1:600) and counter‐labeled with DAPI (catalog no. 320858, Advanced Cell Diagnostics, Newark, CA) to visualize nuclei. Control cells were prepared by omitting either primary or secondary antibodies from the incubation solution. The slides were cover slipped with Mowiol Æ 4–88 with 2.5% DABCO and left overnight in the dark at room temperature. The slides were imaged at 100× or 200× using the Leica TCS SP5 confocal microscope (Leica Micro‐systems, CMS GMBH) with custom software (Leica Micro‐systems LAS AF). Images were acquired using a frame size of 512 × 512 pixels and 4‐line averaging was used to remove noise from the image. Images were then imported in ImageJ (http://rsb.info.nih.gov) using the LOCI Bio‐formats plug‐in (University of Wisconsin, Madison).

### 2.11 Total RNA isolation and RT‐PCR analysis

RNA was extracted from the cells using Trizol (catalog no. 15596026, Invitrogen) followed by purification using the RNA Clean and Concentrator Kit (Zymo Research, catalog no. R1013). The RNA (1 μg) was converted to complementary DNA (cDNA) using iScript Reverse Transcription Supermix (Bio‐Rad, catalog no. 1708840) per the manufacturer's instructions. The cDNA was diluted 1:4 with water. PCR products representing the marker paralogs were detected with a 20 μL PCR reaction mixture containing sequence‐specific primers (Table 1), GoTaq Master Mix (Promega, catalog no. M7132), and a 1/20 volume of cDNA. The mixture was subjected to 35 cycles of amplification (denaturation at 95°C for 30 s, annealing at 56°C for 30 s, and extension at 72°C for 20 s). The amplified DNA was resolved on a 2% agarose gel alongside a 50 bp ladder.

**TABLE 1:** List of primers used for quantitative real‐time PCR analysis.

Alpha fetoprotein	*Afp*	Forward: GATAGCTTCCACGTTAGATTCCT Reverse: GTCATTTTGTTCACTTCCTCCTC
Albumin	*Alb*	Forward: GAAGTGCTCCAGTATGCAGAA Reverse: ACTTTGGTCAGGTCTGTTGC
Anoctamin 1, calcium activated chloride channel	*Ano1 (Tmem16a)*	Forward: AAGTTTGTCACCGAGCTACG Reverse: GATGAAGTCAGACGTGAAGGAG
Collagen, type I, alpha 1	*Col1a1*	Forward: CGCAAAGAGTCTACATGTCTAGG Reverse: CATTGTGTATGCAGCTGACTTC
Cystic fibrosis transmembrane conductance regulator	*Cftr*	Forward: GTCAAAGCTTGCCAACTACAG Reverse: GCTCTTGCTAAAGAAATCCTTGC
Epithelial cell adhesion molecule	*Epcam*	Forward: TGGTGTCATTAGCAGTCATCG Reverse: GGCATTAAGCTCTCTGTGGAT
Gamma‐glutamyl transferase	*Ggt1*	Forward: CTGACGTATCACCGTATCGTG Reverse: AGAACTCAGAGCTCATGTTGC
Hepatocyte nuclear factor‐1‐beta	Hnf1b	Forward: CACCTCTCTCAACACCTCAAC Reverse: CTGGACTGTCTGGTTGAACTG
Hepatocyte nuclear factor 4 alpha	Hnf4a	Forward: TCCAGTTCATCAAGCTCTTCG Reverse: TGTTCTTGCATCAGGTGAGG
Keratin 7	*Krt7*	Forward: CTGAGAATGAGTTTGTGTTGCTG Reverse: TGAAGGGTCTTGAGGAAGTTG
Keratin 19	*Krt19*	Forward: CTCCCGAGATTACAACCACTAC Reverse: CGAGCATTGTCAATCTGTAGGA
Leucine‐rich repeat‐containing protein 8A	*Lrrc8a*	Forward: CACAACAACCTGACCTTCCTC Reverse: CCGTAGCTTCCGACACTG
SRY‐Box Transcription Factor 9	*Sox9*	Forward: CGACCCATGAACGCCTT Reverse: GTCTCTTCTCGCTCTCGTTC
TATA box binding protein	*Tbp*	Forward: TGTATCTACCGTGAATCTTGGC Reverse: CCAGAACTGAAAATCAACGCAG
Transient receptor potential cation channel subfamily V Member 4	*Trpv4*	Forward: CTGGAGATCCTGGTGTACAAC Reverse: GACCACGTTGATGTAGAAGGAC

### 2.12 Western blot

Total protein extracts were prepared from mouse cholangiocytes as follows: Cells were washed twice with ice‐cold PBS and lysed at 4°C with RIPA buffer (Thermo scientific, catalog no. 89900). Protein fractions were subjected to 7.5% SDS–polyacrylamide gel and transferred to nitrocellulose membranes. After blocking, immunoblots were incubated overnight with anti‐mouse CFTR, 1:1000 (CFF, CFTR217). This was followed by incubation with peroxidase‐conjugated goat anti‐rabbit antibody (1:10,000 dilution, Jackson Immunoresearch Laboratories, Inc., catalog no. 111‐035‐003) and visualized by the ECL^+^ detection kit (GE Healthcare, England, catalog no. RPN2209). Primary antibodies used were CK19 (10712‐1‐AP, Proteintech, 1:500), CK7 (SC‐23876, Santa Cruz Biotechnology, Inc. Dallas, TX, 1:500), CFTR (Ab‐737) (8B0860, Syd labs, Hopkinton, MA, 1:200), and TMEM16A/ANO1(H‐41) (SC‐135235, Santa Cruz, 1:500).

### 2.13 Statistics

Data are presented as the mean ± standard deviation, with *n* representing the number of culture plates or repetitions for each assay as indicated. Statistical analysis included Fisher's paired and unpaired *t*‐test and ANOVA for multiple comparisons to assess statistical significance as indicated, and *p* < 0.05 were considered to be statistically significant.

## 3 RESULTS

### 3.1 Modified culture conditions allow cholangiocyte differentiation and long‐term expansion

Cholangiocytes were isolated from 5 wild‐type (WT) mice and 2 CFTR knockout mice as described in the Methods section. This preparation included collagenase digestion, culturing bile duct sections in collagen for 1 week, and immune affinity separation. To support long‐term growth and differentiation of these purified cholangiocytes, we then cultured them in media that consisted of 50% H69 medium and 50% conditioned medium (CM) obtained from 3T3‐J2 cells cultured in H69 media for 3 days. The media was subsequently supplemented with ROCK inhibitor Y‐27632. The culturing method was used to create successful cell lines from 2 WT mice and 1 CFTR knockout mouse. For consistency, we only used one WT cell line to obtain the morphological and functional data presented in this report.

**FIGURE 1:**
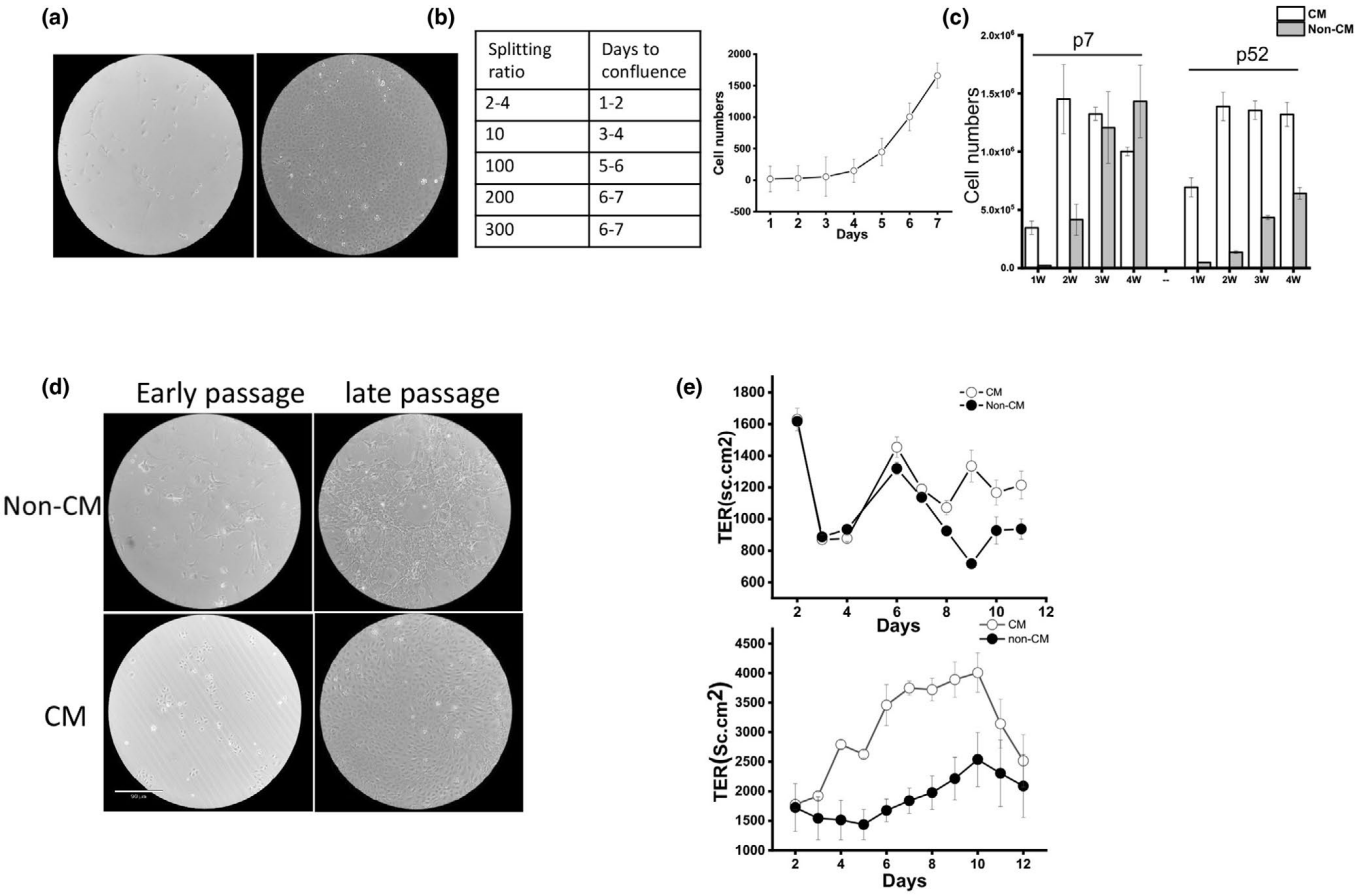
Characterization of normal mouse cholangiocyte (NMC) in culture. (a) Brightfield images of NMC cells treated with conditioned medium (CM) and non‐CM. (b) Cell growth table (splitting ratio) and cell growth curve for NMC in CM; *n* = 3 technical replicates. (c) Quantification of cell number in early versus late passage and CM versus non‐CM; *n* = 3 technical replicates per group. (d) Brightfield images of NMC cells in early versus late passage and CM versus non‐CM. (e) Measurement of trans‐epithelial resistance (TER) in NMC grown in CM versus non‐CM (upper panel), and after removal of CM at day 13 (lower panel); *n* = 4 technical replicates per group. Data is reported as mean ± SD.

Although the cells grew slowly for several days, after 7 days in culture with CM many colonies of epithelial cells were visible, with one cell colony giving rise to hundreds of cells (Figure 1a). In parallel, cells were cultured in non‐CM medium (H69 medium only without 3T3 conditioning or ROCK inhibitor). These cells grew much slower, with a doubling time greater than 1 week. Additionally, these cells acquired a squamous morphology, in contrast to cells grown in CM, which retained an epithelial cell morphology and eventually formed a confluent monolayer.

In the first week in culture, cells growing on plastic showed signs of senescence and had begun to form islands of spreading cells. This could be reversed by stimulation with CM, which induced differentiation and proliferation. Even at high splitting ratios, the cells attained confluency within 7 days (Figure 1b). However, if CM were removed, cell growth slowed. This is demonstrated in Figure 1e, which shows that cell growth in CM was comparable to non‐CM in the beginning but began to diverge after 7 days (upper panel). Trans epithelial resistance (TER), a measure of the strength of tight junctions (Fiorotto et al., 2016) is increased in CM compared to non‐CM. If CM is removed after 13 days, the cell growth again diverges (lower panel), demonstrating that CM media facilitate cell growth and stronger cell–cell tight junctions.

Next, we assessed the replicative capacity of these cells over time under different conditions. NMC at passage 7 and passage 52 showed similar seeding efficiencies after subculture but grew at significantly different rates over a period of 4 weeks. In passage 7, the cells without CM grew much slower than the culture with CM in the first 2 weeks. However, the cell numbers rapidly caught up and were equivalent in both CM and non‐CM by the third and fourth week (Figure 1c). This implies that if cells are allowed to differentiate in the early passages, they can grow indefinitely. However, after 52 passages, cells cultured in non‐CM did not catch up to the number of cells with CM even after 4 weeks (Figure 1c). Light microscopy shows that cultured NMC were composed of uniformly sized, tightly packed, polygonal cells with closely apposed intercellular membranes. Within 2 weeks, the initial colonies had enlarged to form confluent monolayers composed of cells with a uniform morphology. At later passages, however, without CM the cultures began to display increasing heterogeneity in size, degree of spreading, and surface granularity (Figure 1d). Using CM, we have established 2 long‐term NMC cultures, designated NMC‐a and NMC‐b according to the sequence in which they were initiated. The NMC‐a line has been passaged 50 times and maintained in vitro for more than 14 months. Together, these results show that CM stimulates NMC proliferation over time, and that these cells maintain their morphology and growth advantage compared to cells grown in non‐CM.

### 3.2 Normal mouse cholangiocytes (NMC) express and retain markers of differentiated biliary epithelium over time

**FIGURE 2:**
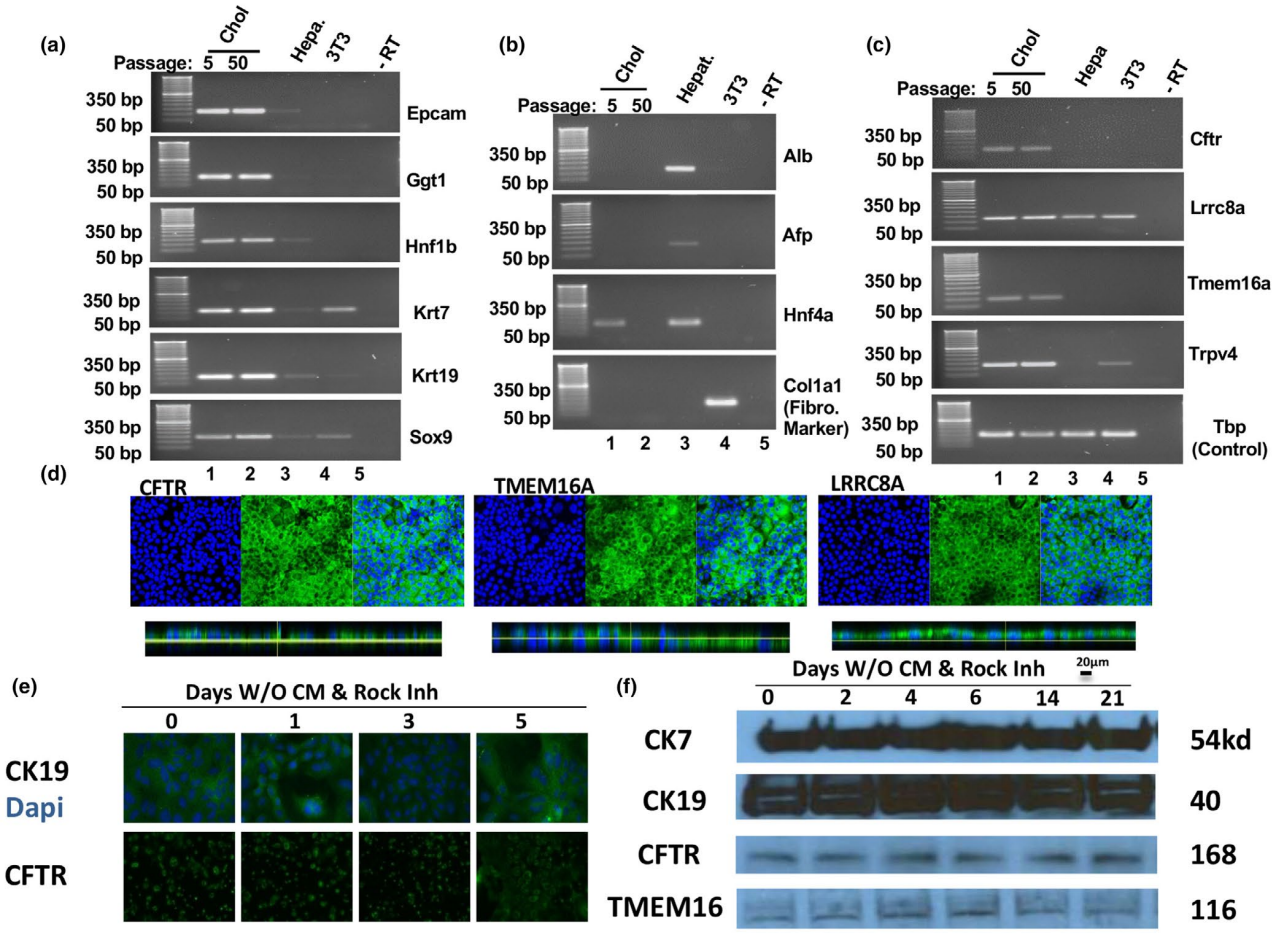
Analysis of cholangiocyte marker expression in NMC. (a) RT‐PCR for cholangiocyte genes (*Epcam, Ggt1, Hnf1b, Krt7, Krt19, Sox9*) in NMC cultured for 5 or 50 passages, primary hepatocytes, and 3T3 cells. RT lane = no reverse transcriptase (negative control). (b) RT‐PCR for hepatocyte and fibrosis genes (*Alb, Afp, Hnf4a, Col1a1*) in NMC cultured for 5 or 50 passages, primary hepatocytes, and 3T3 cells. (c) RT‐PCR for cholangiocyte transporters (*Cftr, Lrrc8a, Tmem16a, Trpv4*) in NMC cultured for 5 or 50 passages, primary hepatocytes, and 3T3 cells. (d) Immunostaining for three Cl‐ channels (CFTR, TMEM16A, LRRC8A) on polarized NMC (100× magnification). (e) CK19 immunostaining on NMC cultured in CM and non‐CM (200× magnification). (f) Comparison of cholangiocyte markers C7, CK19, CFTR, and TMEM16A in NMC cultured in CM and non‐CM by Western blot. One representative well was harvested for protein at the indicated time point to represent the effect of CM removal in the NMC cell line over time.

Cholangiocyte genes *Epcam*, *Ggt1*, *Hnf1β*, *Krt7*, *Krt19*, *Sox9* were expressed in both early and late passages but were absent in hepatocyte cultures (Figure 2a). In contrast, cholangiocytes do not express hepatocyte markers *Alb*, *Afp*, and *Hnf4a*, or fibroblast marker *Col1a1* (Figure 2b). We next examined the expression of the Cl‐ channel CFTR, which is restricted to cholangiocytes, as well as the Cl‐ channels LRRC8A and TMEM16A and the Ca2+ channel TRPV4, which are expressed in cholangiocytes as well as other cell types. As shown in Figure 2c, NMC expressed all three different Cl‐ channels as well as TRPV4. This finding was confirmed by immunostaining, which revealed CFTR, TMEM16A, and LRRC8A on the plasma membrane (Figure 2d). Removal of CM did not reduce expression of biliary marker CK19 (Figure 2e). Furthermore, Western blotting of whole‐cell lysates from NMC harvested at various time points after removal of CM shows that expression of CK7, CK19, CFTR, and TMEM16A remains consistent over time (Figure 2f). Therefore, NMC express cholangiocyte markers after isolation when cultured with or without CM, as well as in long‐term culture.

### 3.3 NMC retain their biophysical properties and Cl‐ conductances in culture

To assess the retention of biophysical properties, electrophysiological techniques are commonly employed. Patch‐clamp recordings can be performed to measure ion currents across the cell membrane, including chloride currents. This allows the evaluation of the specific ion channel activities and the biophysical properties of the cholangiocytes in culture. Cells from the same line used for morphological and marker analysis were used for each of the patch clamp studies described below. Cells were analyzed from different plates on multiple dates, and measurements were repeated on at least three different days.

#### 3.3.1 CFTR Cl‐ currents

Cystic fibrosis transmembrane conductance regulator (CFTR) is a Cl‐ channel expressed on the apical surface of cholangiocytes that is activated by cAMP/PKA. (Tabibian et al., 2013) Unlike other Cl‐ channels, CFTR is only expressed on cholangiocytes, not on all liver cells; thus, we sought to establish CFTR function as a primary indicator of successful long‐term cholangiocyte culture. To assess the functionality of CFTR channels in NMC cells, membrane currents were measured via whole cell patch‐clamp techniques. Cells on a coverslip were mounted in a chamber and currents were measured with a standard CFTR extracellular solution (see Methods section). Cells were then exposed to 10uM forskolin+10uM isobutyl methylxanthine (IBMX), which induced current typical of CFTR activation. As shown, the current–voltage relationship was linear, demonstrating that NMC cells exhibit cAMP‐stimulated Cl‐currents.

**FIGURE 3:**
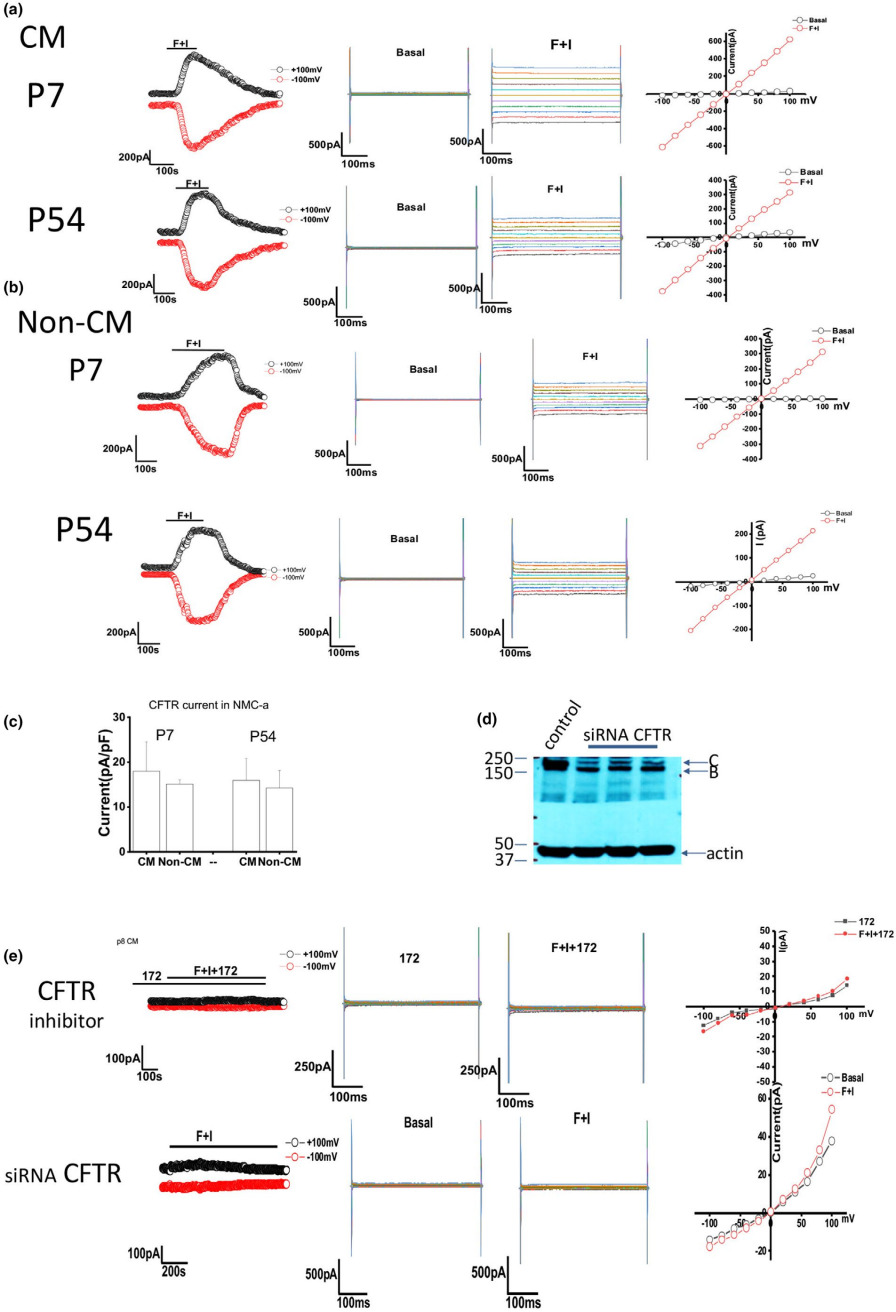
Measurement of CFTR Cl‐ currents in NMC. (a, b) Left: Whole cell patch clamp studies in NMC exposed to 100 μM forskolin and 10 μM IBMX. Currents measured at −100 mV (bottom red circles) and at +100 mV (top black circles) are shown. Middle: Currents were measured in NMC during basal (control) conditions and in response to forskolin and IBMX (F + I) using STEP protocol. Right: Current–voltage (I‐V) plot generated by STEP protocol. (a) NMC cultured in CM. Top: NMC from passage 7; bottom: NMC from passage 54. (b) NMC cultured in non‐CM. Top: NMC from passage 7; bottom: NMC from passage 54. (c) Summary of CFTR currents in early passage versus late passage and CM versus non‐CM. Data reflects the total number of cells measured from three technical replicates. For CM p7 *n* = 13 cell currents were measured; for non‐CM p7 *n* = 9; for CM p54 *n* = 8; for non‐CM p54 *n* = 8. (d) Western blot shows successful knockdown of the CFTR C band after treating NMC with CFTR siRNA. (e) Whole cell patch clamp studies in NMC treated with either CFTR inhibitor 172 (top), or transfected with CFTR siRNA (bottom) and exposed to 100 μM forskolin and 10 μM IBMX. Data for E is reported as mean −/+ SD.

We then compared early passage (p7) and later passage (p50) NMC with and without CM (Figure 3a,b). The results show that there is no significant difference between early passage (*n* = 9) and late passage (*n* = 15) in forskolin cocktail‐induced Cl‐ current. Additionally, there is no significant difference between the cultures with CM (*n* = 12) and without CM (*n* = 11) (Figure 3c). The average CFTR current value was between 10 and 20 pA/pF in all groups. We therefore concluded that the cells exhibit CFTR functionality regardless of the number of passages and despite the removal of CM.

To strengthen our findings, CFTR specific inhibitor 172 was used to verify the forskolin cocktail activated current. (Fiorotto et al., 2016) When NMC cells were preincubated with 172 from passage 12 to passage 15, CFTR currents were remarkably inhibited (Figure 3e). NMC cells were transfected with siRNA against CFTR, which knocks down the mature, complex‐glycosylated C band, leaving the core glycosylated form of the protein (B band) intact (Patrick et al., 2011) (Figure 3d). No currents were detected in NMC after knockdown of CFTR (Figure 3e), confirming that the Cl‐ currents were a result of CFTR activation.

#### 3.3.2 ATP‐stimulated currents

**FIGURE 4:**
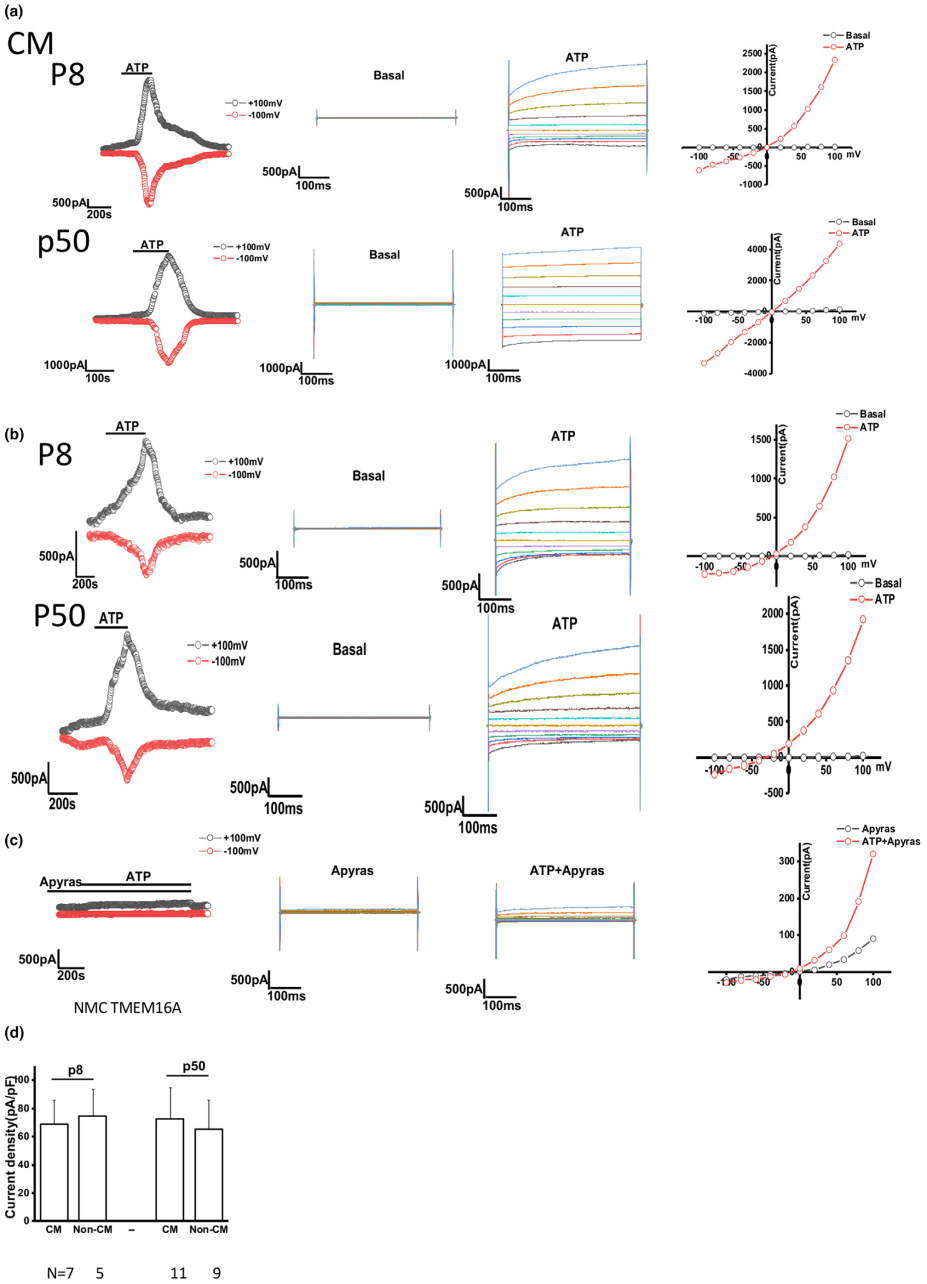
Measurement of the TMEM16A‐mediated ATP activated Cl‐ current in NMC. (a–c) Left: Whole cell patch clamp studies in NMC exposed to 100 μM ATP. Currents measured at −100 mV (bottom red circles) and at +100 mV (top black circles) are shown. Middle: Currents were measured in NMC during basal (control) conditions and in response to ATP using STEP protocol. Right: Current–voltage (I‐V) plot generated by STEP protocol. (a) NMC cultured in CM. Top: NMC from passage 8; bottom: NMC from passage 50. (b) NMC cultured in non‐CM. Top: NMC from passage 8; bottom: NMC from passage 50. (c) NMC treated with apyrase, which hydrolyzes ATP. (d) Summary of TMEM16A currents in early passage versus late passage and CM versus non‐CM. Data reflects the total number of cells measured from three technical replicates. For CM p8 *n* = 7 cell currents were measured; for non‐CM p8 *n* = 5; for CM p50 *n* = 11; for non‐CM p50 *n* = 9. Data for D is reported as mean −/+ SD.

Transmembrane member 16A protein (TMEM16A) is a Ca2+ −activated Cl‐ channel that is expressed on the cholangiocyte plasma membrane (Dutta et al., 2011) Expression of TMEM16A increases transepithelial secretion in response to extracellular nucleotides. To determine the functionality of TMEM16A in NMC, we measured whole‐cell currents using patch clamping. Exposure of NMC cells to ATP resulted in instantaneous activation of Cl‐ currents with time‐dependent activation at positive potentials (greater than +60 mV), outward rectification, and reversal at 0 mV (*E*_Cl_^−^ = 0), consistent with the properties of TMEM16A. In some cases, the ATP‐stimulated currents demonstrated an oscillatory pattern. Thus, isolated primary cholangiocytes express TMEM16A and exhibit Ca2+ −activated Cl‐ currents in response to ATP. We did not observe any significant difference between current measured in early passage cells (p8) and late passage (p50) cells (Figure 4a,d). Additionally, culturing NMC in non‐CM does not affect the function of TMEM16A (Figure 4b,d). Finally, ATP activated current was abolished by apyrase, which hydrolyzes ATP (Figure 4c), demonstrating the specificity of this response.

#### 3.3.3 Volume‐stimulated Cl‐ currents

**FIGURE 5:**
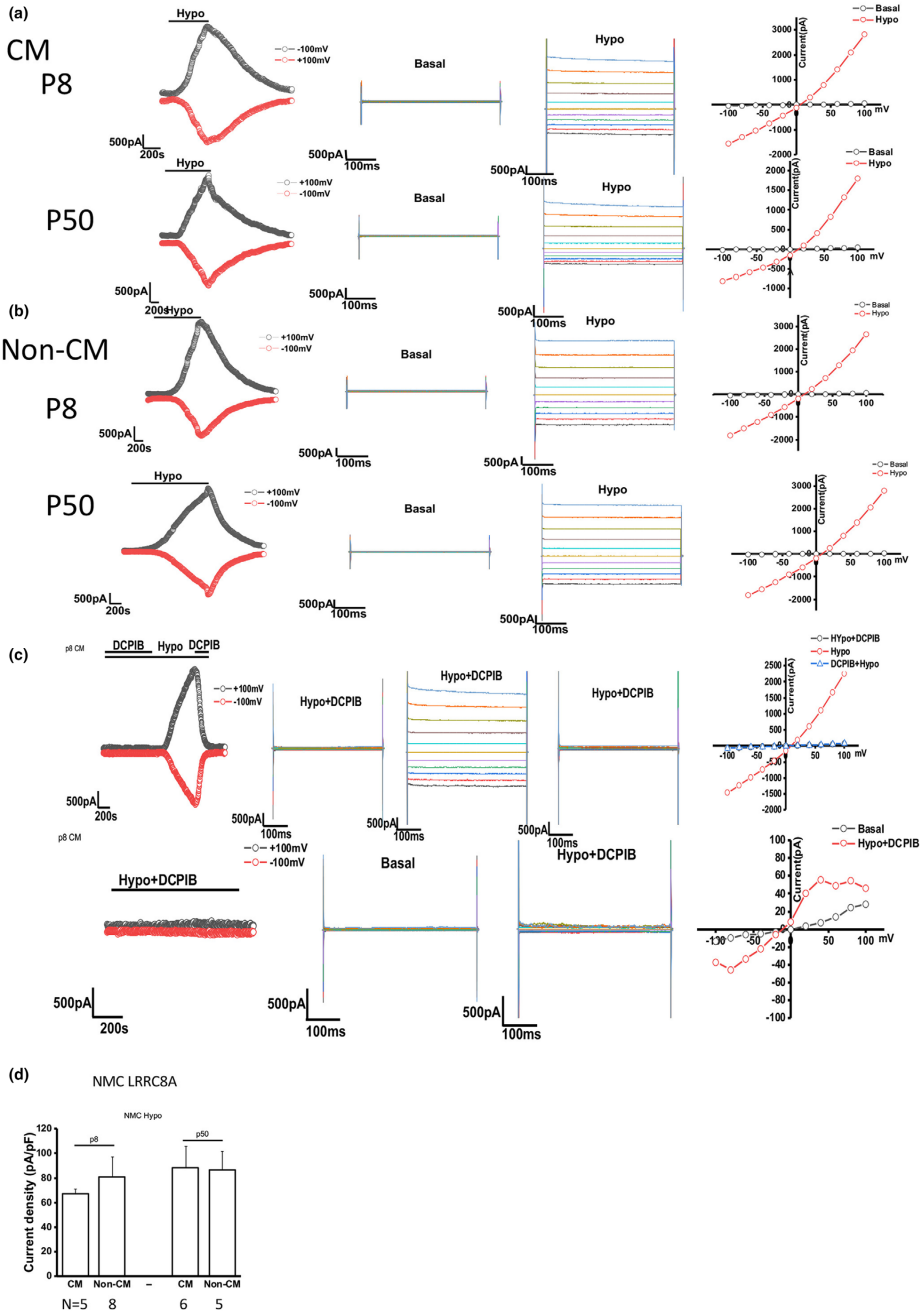
Measurement of the LRRC8A‐mediated volume‐stimulated Cl‐ current in NMC. (a–c) Left: Whole cell patch clamp studies in NMC exposed to 33% hypotonic solution. Currents measured at −100 mV (bottom red circles) and at +100 mV (top black circles) are shown. Middle: Currents were measured in NMC during basal (control) conditions and in response to 33% hypotonic solution using STEP protocol. Right: Current–voltage (I‐V) plot generated by STEP protocol. (a) NMC cultured in CM. Top: NMC from passage 8; bottom: NMC from passage 50. (b) NMC cultured in non‐CM. Top: NMC from passage 8; bottom: NMC from passage 50. (c) NMC treated with DCPIB, an inhibitor specific for LRRC8A. (d) Summary of LRRC8A currents in early passage versus late passage and CM versus non‐CM. Data reflects the total number of cells measured from three technical replicates. For CM p8 *n* = 5 cell currents were measured; for non‐CM p8 *n* = 8; for CM p50 *n* = 6; for non‐CM p50 *n* = 5. Data for D is reported as mean −/+ SD.

Volume‐stimulated Cl‐ channels such as leucine rich repeat‐containing 8 subunit A (LRRC8A) are present in all cells and are responsive to cell swelling and increased volume (Voss et al., 2014) To determine how changes in cell volume affect these channels in NMC, the cells were exposed to a 33% hypotonic solution. Under basal conditions with standard intra‐ and extracellular buffers, *I*_Cl_ was small (−0.8 ± 0.2 pA/pF). Exposure to 33% hypotonic solution resulted in activation of currents within 4–6 min, increasing current density to −14.0 ± 0.9 pA/pF at −80 mV. The currents measured at 0 mV were transient and of small magnitude, while the currents measured at −80 mV were sustained for the duration of hypotonic exposure and were fully reversible within 10 min of isotonic solution. These large volume‐stimulated currents exhibited reversal near −15 mV (*E*_Cl_), outward rectification, and time‐dependent inactivation at depolarizing potentials above +60 mV, characteristics different from ATP‐stimulated Cl^−^ currents previously described in these cells (Figure 5a). The magnitude of volume‐stimulated currents was 3‐fold greater than the currents stimulated by cAMP (CFTR current). There was no significant difference between the cells cultured with CM or in non‐CM. Similar results were also obtained from early passage and late passage NMC (Figure 5a,b,d). Finally, treatment with LRRC8A specific inhibitor DCPIB completely abolished current activity (Figure 5c). These results confirm that NMC respond to hypotonic conditions and produce volume‐stimulated currents regardless of culturing conditions and number of passages.

### 3.4 Cholangiocytes from CFTR knock out mouse (CFMC) lack CFTR current but otherwise display similar biophysical properties to NMC

**FIGURE 6:**
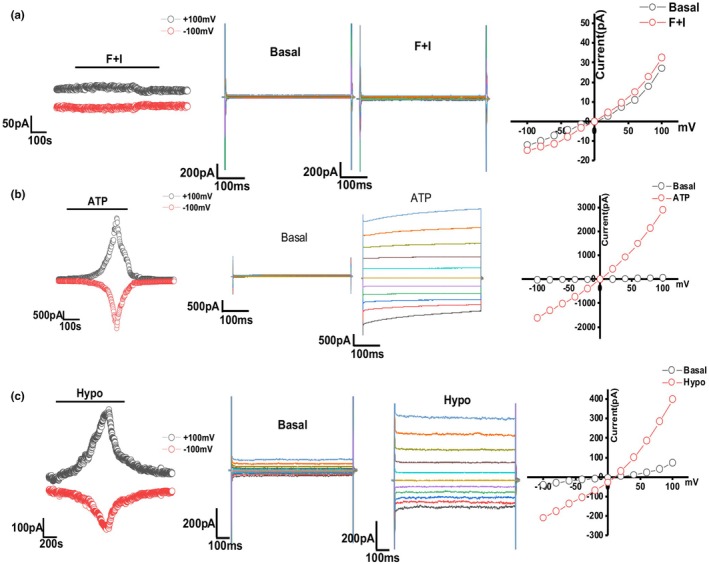
Measurement of three different Cl‐ channels in cholangiocytes from CFTR knock out mouse (CFMC). (a–c) Left: Whole cell patch clamp studies. Currents measured at −100 mV (bottom red circles) and at +100 mV (top black circles) are shown. Middle: Currents were measured in NMC during basal (control) conditions and in response to an activator (F + I, ATP, or hypotonic buffer) using STEP protocol. Right: Current–voltage (I‐V) plot generated by STEP protocol. (a) CFMC exposed to 100 μM forskolin and 10 μM IBMX. (b) CFMC exposed to 100 μM ATP. (c) CFMC exposed to 33% hypotonic solution. Data are representative of measurements from three technical replicates.

To further validate our culture methods, we isolated cholangiocytes from CFTR knockout mice using the same methods used for NMC. We successfully established a CFTR knockout cholangiocyte cell line and tested the activity of the three different Cl‐ channel activities. As expected, CFTR currents were absent when CFMC cells were exposed to forskolin cocktail (Figure 6a). However, TMEM16A currents were maintained (Figure 6b), and LRRC8A currents were also recorded after application of hypotonic solution (Figure 6c). Therefore, while CFTR currents were abolished in CFMC due to loss of CFTR protein, all other currents were present. These studies indicate that our protocol is capable of maintaining both normal and genetically mutated cholangiocytes in culture.

### 3.5 Both NMC and CFMC release ATP, resulting in increased intracellular Ca2+

**FIGURE 7:**
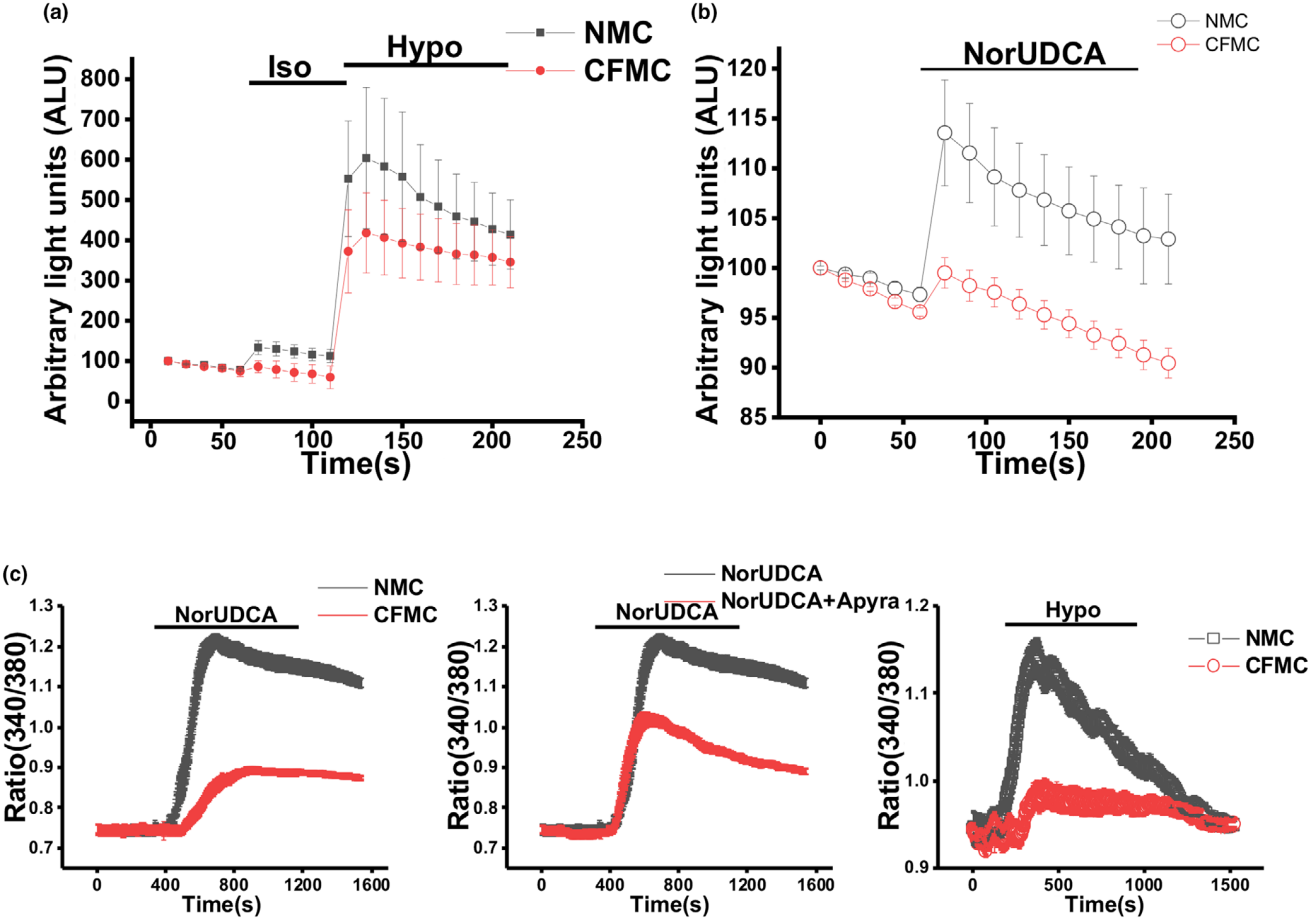
Measurement of ATP release and changes in intracellular Ca2+ in NMC and CFMC. (a) Hypotonic stress‐induced ATP release from NMC (black circles) and CFMC (red circles). ATP in the extracellular media was detected using the luciferin‐luciferase assay and quantified as arbitrary light units (ALU). The y‐axis represents relative increase from basal luminescence (expressed as relative ALU/μg/mL protein) after addition of 33% hypotonic media. (b) NorUDCA‐induced activation of TMEM16A channels in NMC (black circles) and CFMC (red circles). As in (a), ATP release is quantified as ALU. (c) Intracellular Ca2+ changes induced by NorUDCA and 33% hypotonic media. Left: Comparison of Ca2+ accumulation in NMC (black) and CFMC (red) after treatment with NorUDCA. Middle: Comparison of Ca2+ accumulation in NMC (black) and NMC + ATP inhibitor apyrase (red) after treatment with NorUDCA. Right: Comparison of Ca2+ accumulation in NMC (black) and CFMC (red) after treatment with hypotonic media. Changes in intracellular calcium were plotted as a ratio of calcium‐bound fura‐2 am (340 nm) and calcium‐free fura‐2 am (380 nm). For A and B, *n* = 3 technical replicates per group, and data is reported as mean −/+ SD. For C, studies were conducted on 20 ~ 30 cells per field, and repeated in three technical replicates. Representative images are shown.

To determine whether NMC cells release ATP in response to hypotonic solution, ATP in the extracellular media was detected using the luciferin‐luciferase assay and quantified as arbitrary light units (ALU). Exposing the NMC to 33% hypotonic media resulted in a rapid increase in ATP release that occurred within seconds and was significantly increased over control cells exposed to isotonic exposure. Although the CFMC also responded to hypotonic solution, ATP release was decreased compared to the NMC (Figure 7a). 24‐norUrsodeoxycholic acid (norUDCA), a synthetic analog of the hydrophilic bile acid UDCA, can induce bile acid elimination by stimulating Cl‐ channels. NorUDCA can also stimulate ATP release. Figure 7b shows that ATP release increased when cells were exposed to norUDCA. However, NMC cells released more ATP than norUDCA‐treated CFMC. The decreased responsiveness to both hypotonic solution and norUDCA confirms CFTR's significant contribution to ATP release.

ATP release increases intracellular Ca2+, a key step in regulating ductular secretion and Cl‐ efflux (Li et al., 2018) Hypotonic solution and norUDCA increased intracellular Ca2+ in NMC, which was measured by fura‐2 am (Figure 7c). The specificity of this response was confirmed by treatment with apyrase, which hydrolyzes ATP and thus inhibits the increase of intracellular Ca2+. Parallel experiments with NMC and CFMC showed that intracellular Ca2+ levels were blunted when CFMC were treated with norUDCA or hypotonic solution (Figure 7c). Together, these studies demonstrate that functional CFTR in the cholangiocyte membrane is required for ATP release and accumulation of intracellular Ca2+.

## 4 DISCUSSION

Cholangiocytes, which form the lining of the biliary tract, play an essential role in bile formation and secretion in the liver. These cells, which are heterogenous in structure and function, are polarized, possessing an apical and a basolateral plasma membrane. Under physiological conditions, cholangiocytes modulate bile in the ductal lumen through transport of Cl‐, HCO_3_‐, and water across the apical membrane, which results in alkalinization of bile and increased bile volume (Boyer, 2013; Tabibian et al., 2013) This process is driven by increases in cAMP and Ca2+. Cholangiocytes also contribute to bile modification through absorption of ions, bile acids, glucose, and other molecules. Notably, cholangiocytes are both a target of and a participant in various disease states where bile flow is impeded or decreased (Banales et al., 2019) Despite their importance in both physiology and pathophysiology, the use of primary cholangiocytes for in vitro studies has been limited in part by difficulties in isolating these cells from the intrahepatic mass. Long‐term culture of cholangiocytes from normal liver has also been hampered by the relatively small numbers of ductal cells obtained from isolation and by complex culture procedures (Kudira et al., 2021; Luce & Dubart‐Kupperschmitt, 2020) Thus, hyperplastic or immortalized cholangiocytes have been widely used as a surrogate (Sirica et al., 1990; Ueno et al., 2003) These cells have been well characterized; however, constitutively proliferating cells can acquire alterations in gene expression and phenotype over time that may affect their physiological function. To avoid these pitfalls, we have developed a protocol for establishing primary cultures of NMC from normal adult mouse liver that allows for long‐term maintenance of primary cholangiocytes in culture.

Previously, normal rat cholangiocytes (NRC) were shown to retain many of the phenotypic features of cholangiocytes in situ when cultured in the presence of collagen and defined media supplements, including the ability to form monolayers (Vroman & LaRusso, 1996) NRC have since been utilized in transport and electrophysiologic studies to characterize glucose transport, Na + ‐dependent bile acid transport, and cAMP‐stimulated Cl‐ secretion (Lazaridis, Pham, Tietz, et al., 1997; Lazaridis, Pham, Vroman, et al., 1997; Salter et al., 2000; Spirli et al., 1998) By replicating these conditions in the preparation of our primary mouse cell lines, we were able to obtain similar findings and demonstrate the utility of NMC cells as a model for investigating the cellular mechanisms responsible for cholangiocyte secretion and bile formation.

To isolate cholangiocytes, we first precultured intact bile duct fragments in a collagen gel for 7 days to reproduce the methodology used for isolating NRC (Yang et al., 1993) This technique was thought to be superior to previous protocols that use elutriation and antibody‐coated magnetic beads to isolate cholangiocytes from enzymatically dissociated biliary trees because cholangiocytes can survive and proliferate within the ducts due to maintenance of cell contacts in ductal fragments (Sirica et al., 1990; Yang et al., 1993) We employed separation and purification of cholangiocytes with EpCAM antibody only after the preculturing phase was complete. The role of extracellular matrix in maintaining cell shape, proliferation, and differentiation of epithelial cells is well known (Daley et al., 2008) Our results using collagen gels seemed to confirm this. However, we could also bypass this step by dicing bile ducts into small pieces and using trypsin and DNAse to obtain single cell suspensions. Results from preliminary studies suggest that the growth and efficiency of bile duct epithelial cells were not significantly different if first preincubated in collagen gels or cultured directly after enzymatic digestion.

Y‐27632 is a selective inhibitor of Rho kinase (ROCK), which is involved in a variety of cellular processes, including contraction, adhesion, migration, proliferation, and apoptosis through its regulation of actin cytoskeleton assembly and cell contraction (Riento & Ridley, 2003; Saadeldin et al., 2020) Y‐27632 was initially shown to increase the cloning efficiency of human embryonic stem cells, enhancing their survival by preventing apoptosis (Watanabe et al., 2007) Since then, Y‐27632 has been used to regulate the differentiation and proliferation of multiple types of stem cells (Joo et al., 2012; Kurosawa, 2012) Notably, Y‐27632 also induced indefinite cell proliferation of primary human keratinocytes in culture (Chapman et al., 2010; van den Bogaard et al., 2012) This process, which is referred to as conditional immortalization or conditional reprogramming, produces conditionally reprogrammed cells (CRC) without the need for a feeder layer. Supplementing media with Y‐27632 enables unlimited, feeder‐free expansion of porcine airway epithelial cells while maintaining an epithelial phenotype (Dale et al., 2019) Another technique to extend replicative capacity is the treatment of ROCK inhibitor in combination with conditioned medium. Medium that has been conditioned by exposure to fibroblasts such as the 3T3‐J2 cell line used in our experiments contains secreted paracrine factors that can maintain cell viability and function (Jeong et al., 2016) The combination of Y‐27632 and feeder cells has been successfully used to convert multiple epithelial cell types to a proliferative state while simultaneously maintaining a normal cellular phenotype (Liu et al., 2012) Bone marrow mesenchymal stem cell differentiation into keratinocytes was also achieved when these cells were exposed to Y‐27632 and keratinocyte CM (Li et al., 2015) In our study, primary NMC had not undergone senescence even after 50 passages in the presence of both Y‐27632 and CM, demonstrating the efficacy of combining a proliferative enhancer with stromal‐derived paracrine signals that maintain physiological function.

Phenotypically, NMC display a phenotype consistent with a biliary origin, with a small diameter and an epithelial morphology. The colony formation assay also demonstrated that both Y‐27632 and CM are required for optimal growth of the largest and most numerous cholangiocytes, clones with normal epithelial morphology. Under these culture conditions, cholangiocytes maintained expression of differentiated biliary markers after 52 passages and 14 months in culture. The retention of biophysical properties in cultured cholangiocytes can be influenced by the duration of culture, passage number, and culture environment. However, in NMC, the presence of these channels and the subsequent confirmation of their function by electrophysiological techniques indicate that these factors were not impacted by long‐term culture. Importantly, when cultured in non‐CM, NMC retain many of the same differentiation markers and functions as cells cultured in CM, albeit with slower growth kinetics. Although NMC need CM in early passages for expansion, once a cell line has been established, it can be maintained in non‐CM, which reduces the time, effort, and costs associated with standard conditional reprogramming protocols.

The goal of this study was to determine isolation and culture conditions that not only extend in vitro life span but preserve differentiation capacity, including CFTR function. Human bronchial epithelial basal cells (HBECs) gradually lose CFTR function over time (Gentzsch et al., 2017) Modifying the standard CRC protocol allowed for the long‐term growth of both normal and cystic fibrosis (CF) HBECs while maintaining their capacity for differentiation (Peters‐Hall et al., 2018) Similarly, we were able to extend the life span of CF and non‐CF bile duct epithelial cells and maintain primary‐like cell characteristics, including multipotent differentiation potential and CFTR expression. We also demonstrated experimentally that NMC conduct Ca2+ activated and volume‐stimulated Cl‐ membrane currents, and that other physiological functions, such as ATP release and accumulation of intracellular Ca2+, are preserved as well. We thus conclude that these cells more accurately reflect in situ cholangiocytes than other cells previously used to study CF that were altered by expression of viral oncogenes or telomerase.

There are important limitations to the current study. First, we utilized the same cell line for all functional analyses in order to maintain consistency. However, performing all analyses in NMC from a single animal does not allow for the assessment of biological variability and raises the issue of pseudoreplication, since the experimental replicates are technical repetitions of the same culture. Future studies utilizing additional animals and strains will be important to demonstrate reproducibility of the protocol. Second, the seeding density for the NMC cultures was low, ranging from one cell to several hundred. This is partly attributable to low yields, since cholangiocytes represent only 3%–5% of the total cell mass (Banales et al., 2019) The numbers were further reduced after purification with antibody‐conjugated beads in order to get a highly homogeneous population of cholangiocytes. Nonetheless, despite their relative scarcity, primary cholangiocytes grew to confluence and maintained their proliferative capacity during long‐term culture. Additionally, we were able to successfully produce colonies from single cells and then expand these colonies to create cell lines, demonstrating clonal ability. Future studies will be needed to determine whether these NMC cell lines undergo genetic drift and/or chromosomal instability, and whether these cells ultimately undergo senescence in the absence of immortalization. The impact of this work would also be enhanced by successfully isolating and culturing human cholangiocytes using these techniques.

Despite multiple studies characterizing the phenotype of cholangiocytes in culture, maintaining these cells in a differentiated state over multiple passages has remained a challenge (Kudira et al., 2021) These limitations have prompted a search for alternative cell sources that are suitable for genetic and functional studies. Previous studies have shown that mouse cholangiocytes isolated from normal mice (BALB/c) and immortalized by transfection with the SV40 large‐T antigen display features of freshly isolated small and large mouse cholangiocytes (Francis et al., 2008; Ueno et al., 2003) Cholangiocytes can also be differentiated from induced pluripotent stem cells into cholangiocyte‐like cells with gene expression profiles and marker expression similar to primary human cells (Florentino et al., 2022; Luce & Dubart‐Kupperschmitt, 2020) Organoids generated from bile ducts retain their original tissue characteristics and can be stably passaged (Chen et al., 2022) Cholangiocytes isolated from diseased tissue may also provide a source of cholangiocytes to study the pathogenesis of cholangiopathies (Tabibian et al., 2014) However, most of these studies lack a detailed assessment of transmembrane receptor functionality. We have used specialized isolation and culture techniques to maintain primary mouse cholangiocytes that not only retain biophysical properties and distinct Cl‐ conductances but also undergo an extended number of passages. The NMC produced from this reliable protocol should provide a vital tool that will advance our understanding of cholangiocyte physiology and pathobiology.

## 5 CONCLUSION

The cell preparation techniques described in this report allow for the isolation of viable cholangiocytes from mouse liver. Our culturing methods further optimize survival and maintain differentiation of these cells in long‐term culture. Functional studies on cells obtained using this protocol illustrate the application of the method in a physiological research context, and demonstrate the stability of the phenotype over multiple passages. This reliable isolation and culture protocol may be useful for future functional analysis, biochemical studies, and cytotoxicity assays.

## AUTHOR CONTRIBUTIONS

**Qin Li, Youxue Wang, Kristy Boggs, Charles Kresge, Kari Nejak‐Bowen:** data curation, formal analysis; **Qin Li, Youxue Wang, Kristy Boggs, Charles Kresge:** investigation; **Qin Li:** conceptualization, methodology, supervision, validation, writing—original draft; **Qin Li, Kari Nejak‐Bowen:** writing—review and edit; **Kari Nejak‐Bowen:** funding acquisition.

## FUNDING INFORMATION

This study was funded by R01DK119435 and R01DK103775 to Kari Nejak‐Bowen, and P30DK120531 to the Pittsburgh Liver Research Center.

## CONFLICTS OF INTEREST

Kari Nejak‐Bowen is a consultant for Surrozen, Inc.

## ETHICS STATEMENT

All animal studies were compliant with ARRIVE guidelines, and performed in accordance with the National Institutes of Healthand the Institutional Animal Use and Care Committee at the University of Pittsburgh School of Medicine (protocol number IS00018809).

## Supporting information


Data S1:

## Data Availability

All data supporting the findings of this study are available within this paper or can be obtained from the corresponding author upon reasonable request.
