# Correction to “Isolation and long‐term culture of primary mouse cholangiocytes that retain biophysical properties and distinct Cl‐conductances: An initial study”

**DOI:** 10.14814/phy2.71021

**Published:** 2026-07-18

Li, Q. , Wang, Y. , Boggs, K. , Kresge, C. , & Nejak‐Bowen, K. (2026). Isolation and long‐term culture of primary mouse cholangiocytes that retain biophysical properties and distinct Cl‐conductances: An initial study. Physiological Reports, 14, e70732. 10.14814/phy2.70732.41559922 PMC12819578

Some errors were noted in the Methods section. In section 2.10 of the Methods (“Immunofluorescence”), the catalog number for the CTFR antibody, ACC‐034 from Alomone Labs, was incorrect.

The correct antibody number for CFTR (Ab‐737) is 8B0860 from Biomol GmbH (Hamburg, Germany).

Likewise, in section 2.12 (“Western blot”), the supplier should be Biomol GmbH instead of Syd labs.

We apologize for these errors which do not affect the scientific conclusions of the study.

