# Supplementary material for: Isolation and long‐term culture of primary mouse cholangiocytes that retain biophysical properties and distinct Cl‐conductances: An initial study
Source: Physiol Rep. 2026 Jan 20;14(2):e70732. doi: 10.14814/phy2.70732 (PMC12819578; doi:10.14814/phy2.70732)
Supplement: Supplementary file 1 — Data S1: [file PHY2-14-e70732-s001.pdf]

# 8921_0_art_file_212226_t55s55.docx

WORD COUNT 9782
TIME SUBMITTED 04-NOV-2025 06:41AM
PAPER ID 118885211

Isolation and long-term culture of primary mouse cholangiocytes that retain biophysical properties and distinct Cl- conductances

Qin Li [1], Youxue Wang[2], Kristy Boggs[1], Charles Kresge[2], Kari Nejak-Bowen[3,4,5]

[1]Department of Pediatrics, University of Pittsburgh Medical Center (UPMC), Pittsburgh, PA; [2]Department of Pediatrics, University of Texas Southwestern Medical Center, Dallas, TX; [3]Organ Pathobiology and Therapeutics Institute, University of Pittsburgh School of Medicine, Pittsburgh, PA, USA, [4]Department of Pharmacology and Chemical Biology, University of Pittsburgh School of Medicine, Pittsburgh, PA, USA, [5]Pittsburgh Liver Research Center, University of Pittsburgh and University of Pittsburgh Medical Center, Pittsburgh, PA, USA,

Running title: Normal mouse cholangiocytes retain Cl- conductance

*Address correspondence to:*

Kari Nejak-Bowen, MBA, PhD
S405A-BST, 200 Lothrop Street
University of Pittsburgh School of Medicine
Pittsburgh, Pennsylvania 15213.
E-mail: knnst5@pitt.edu

Keywords: bile duct epithelial cells, patch clamp, chloride currents, cystic fibrosis transmembrane conductance regulator

Number of figures and tables: 7 figures, 1 table

**Conflict of Interest Statement:** Kari Nejak-Bowen is a consultant for Surrozen, Inc.

**Financial Support Statement:** This study was funded by R01DK119435 and R01DK103775 to Kari Nejak-Bowen, and P30DK120531 to the Pittsburgh Liver Research Center.

**Availability of data and materials:** All data supporting the findings of this study are available within this paper or can be obtained from the corresponding author upon reasonable request.

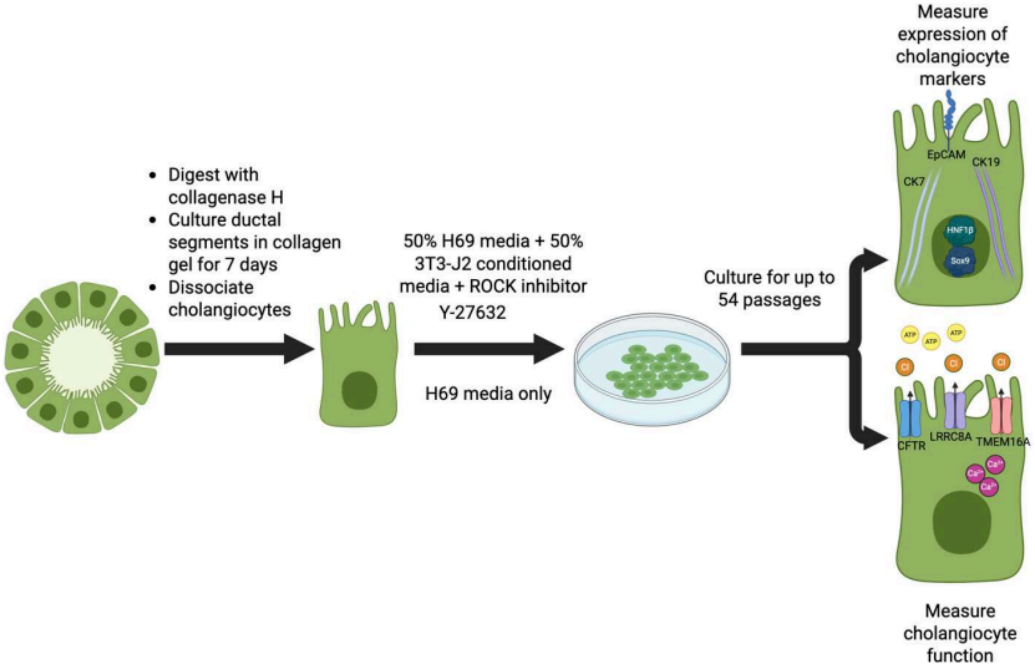


**Graphical Abstract Description**

Mouse cholangiocytes are isolated from bile ducts and cultured in conditioned media containing ROCK inhibitor. Cells maintain expression of biliary markers & ion channel activity for >50 passages.

**Background & Aims:** Cultures of primary mouse bile duct epithelial cells are a valuable tool to study cholangiocyte secretion and bile formation. However, freshly isolated cells have a limited ability to expand in culture. Here we report a novel isolation and culture technique for normal mouse cholangiocytes (NMC) that enables long-term growth without compromising function.

**Methods:** Mouse cholangiocytes were isolated and cultured in conditioned medium (CM) that was subsequently supplemented with ROCK inhibitor Y-27632. Expression of cholangiocyte markers was assessed by qPCR, immunofluorescence, and Western blotting. Patch clamp techniques were used to measure cAMP-activated Cl- current, Ca2+-activated Cl- current, and volume-stimulated Cl- current.

**Results:** Cultures of NMC were polarized and maintained a cholangiocyte phenotype for over 50 passages. Functional studies show that ion channel activity is maintained in NMC regardless of the number of passages and despite removal of CM. NMC also perform other physiological functions such as ATP release and intracellular Ca2+ changes in response to stimulation with bile acids.

**Conclusions:** NMC maintain biophysical properties such as membrane channel expression and secretory functions in long-term culture. These studies demonstrate the utility of NMC cells as a model for investigating the cellular mechanisms responsible for cholangiocyte secretion and bile formation.

## INTRODUCTION

21 Cholangiocytes are epithelial cells that line the intrahepatic bile ducts. The major physiologic function of cholangiocytes is bile formation and transport. 21 Bile formation is a complex process regulated by hormones, peptides, nucleotides, neurotransmitters, and biliary constituents. Cholangiocytes modify 18 primary (canalicular) bile secreted by hepatocytes through a series of secretory and re-absorptive processes. This is achieved by the coordinated vectorial transport of selected ions, solutes, and water across the cholangiocyte apical and basolateral plasma membranes via numerous channels, transporters, and exchangers.(1-3)

While primary fresh isolated mouse cholangiocytes accurately represent the phenotype of cells in vivo, daily isolation and the need for immediate use is a barrier for widespread adoption. Freshly isolated cells have 6 a limited ability to expand in sufficient numbers in culture. Because of this, much of the 16 research in the mouse cholangiocyte field has been carried out with commercially available cell lines with a malignant origin.(4-6) Recently, 35 cholangiocyte-like cells from induced pluripotent stem cells and cholangiocyte-derived organoids have provided new sources of biliary cells for study.(7-10) However, for the most part these protocols prioritized morphological characterization and lacked functional assessment. Methods that achieve 42 long-term culture and cloning of primary mouse cholangiocytes 6 that maintain multipotent differentiation capacity and Cl- channel function would therefore be advantageous.

A recent study showed that conditional reprogramming of cells with a Rho-associated protein kinase (ROCK) inhibitor extended the life span of primary human bronchial epithelial cells.(11) The technique conditionally maintains epithelial cells in a stem cell-like state that enables long-term growth. In the present report, we describe the adaptation of these methods to the long-term culture of normal mouse cholangiocytes (NMC) isolated from mouse liver. Successful isolation and culture techniques have been developed to maintain the viability and functionality of these cells, including the retention of their biophysical properties and distinct chloride (Cl-) conductance. We have explored three different Cl- channels in NMC cells. Our results show that the NMC retain normal physiological function in both early and late passages. These techniques provide a method of maintaining long term cultures of cholangiocytes and should prove to be a valuable tool in the field of biliary biology.

## METHODS

### Animals

A total of 5 wild-type mice and 2 CFTR knockout mice were used for the cell isolation and culturing methods described in detail below. All animal studies were performed following the guidelines of the Institutional Animal Use and Care Committee at the University of Pittsburgh School of Medicine and the National Institutes of Health (Protocol number: IS00018809). All mice were in a C57Bl/6 background (Jackson Laboratories) and maintained in ventilated cages under 12h light/dark cycles with access to enrichment, water, and mouse chow ad libitum.

### Media and solutions.

*Tissue wash solution*: DMEM supplemented with penicillin (100 units/ml), streptomycin (100 units/ml), and amphotericin B (25 ng/ml). *Tissue digestion solution:* Tissue wash solution with collagenase H (1 mg/ml), and hyaluronidase (0.025 mg/ml). *Cell wash solution:* PBS with 0.5 % of bovine serum albumin (BSA) and 2 mM of EDTA.

*H69 culture medium:* DMEM low glucose (50%), DMEM/F12 (50%), with 10% fetal bovine serum (FBS), Penicillin (100 units/ml), and streptomycin (100 units/ml), Adenine HCL (30µg/ml), Insulin (5µg/ml), (±) epinephrine (1µg/ml), Epidermal growth factor (human) (10ng/ml), T3 + T (3,3'5-triiodo-L-thyronine + Apo-transferrin) (2.27ng/ml + 8.34µg/ml), Hydrocortisone (13µg/ml).

*3T3-J2 culture medium:* DMEM with 10% fetal calf serum (FCS), Penicillin (100 units/ml), and streptomycin (100 units/ml).

*Conditioned medium:* 3T3-J2 cells were cultured to 70 – 90 % confluence with 3T3-J2 culture medium. They were then treated with mitomycin (5 µg/ml) for 3 hours. After the

cells were washed with PBS 3 times and with culture medium once, they were cultured with H69 medium for 3 days. Then the culture medium (H69) was collected, filtered through a 0.22 μm filter, and stored at – 80 °C until being used as conditioned medium.

**Reagents.**

The Forskolin and IBMX, UDCA and TUDCA compounds were obtained from LC Laboratories (LC Laboratories, Woburn, MA), GSK1016790A from Santa Cruz (Santa Cruz Biotechnology, Inc. Dallas, TX), and all other reagents were from Sigma-Aldrich (St. Louis, MO).

**Cell isolation**

1. **Bile duct cell isolation:**

Liver lobes were resected 2-5 mm distal to the entry sites of hepatic ducts from 3-month-old male mice weighing approximately 28 grams. The liver lobe capsules were removed, and multiple tears were made in the liver lobes with forceps. The liver tissue was then digested with collagenase H (1 mg/ml) and hyaluronidase (0.025 mg/ml) at room temperature and monitored under a microscope. Parenchymal hepatic tissue and connective tissue were removed with enzyme digestion in combination with physical removal and separation with needles. The duct segments were saved and cultured in H69 medium at 37 °C with 5 % $CO_2$.

After overnight culture, the segments of duct with sealed ends and clear lumen were sliced open and cut into small pieces. The diced ductal tissue was cultured in 0.65 mg/ml collagen gel prepared with PureCol EZgel (Advanced BioMatrix, San Diego, CA) and H69 culture medium. After one week of culture, the collagen gel was digested with

collagenase (1 mg/ml) and dispase (0.2 unit/ml) for 1 hour to dissociate the cholangiocytes. Both the suspended cells and cells that were attached to the bottom of the culture dish were cultured for 2 more days before immune affinity separation.

**2. Bile duct cell immune separation:**

The cells were trypsinized and treated with DNase (0.5 mM) in PBS for 35 minutes. After the cells were washed with PBS, they were treated with fresh DNase (1 mM) for an additional 15 minutes. The cells were then pushed through a 100 µm cell strainer. After centrifuge, the cells were re-suspended in 1 ml of PBS with 0.5 % of bovine serum albumin (BSA) and 2 mM of EDTA and were then incubated with 20 µl of Purified Rat Anti-Mouse CD326/Epithelial Cell Adhesion Molecule (EpCAM) antibody (BD Biosciences, San Jose, CA) at 4 °C on a shaker for 1 hour. After the cells were washed twice, they were resuspended in 240 µl of PBS with 0.5 % BSA and 2 mM EDTA and incubated with 60 µl of Anti-Rat Microbeads (Macs Miltenyi Biotec, Auburn, CA) at 4 °C for 10 minutes. After the cells were washed twice, they were resuspended in 500 µl of PBS with 0.5 % BSA and 2 mM EDTA and applied on an equilibrium column in the magnetic field. After the column was washed 3 times, the magnetic field was removed, and the cells were eluted from the column. The cells were then cultured in H69 medium at a density of one to several hundred, depending on the yield after isolation and magnetic purification.

**3. Long-term expansion under conditional reprogramming conditions:**

Two days after separation, the cells were cultured in H69 medium (66.66 %) with 3T3-J2 cell conditioned medium (33.3 3%) and ROCK inhibitor Y-27632 (5 µM, Enzo, Farmingdale, NJ) for 3 weeks, and then with 50 % H69 medium, 50 % conditioned

medium, and 10 µM Y-27632. The conditioned medium was prepared by treating 70 to 90 % confluent 3T3-J2 (Kerafast, Boston, MA) with mitomycin C (5 µg/ml) for 3 hours, culturing them with H69 medium for 3 additional days, and collecting the culture medium. The conditioned medium was filtered through a 0.22 µm filter and stored at -80°C until use.

4. **Cell Counting:**

Cells harvested 1, 2, 3, and 4 weeks after plating were diluted with 0.2% trypan blue and counted using a hemocytometer. Triplex dishes were analyzed for each time point.

**Measurement of cystic fibrosis transmembrane conductance regulator (CFTR) currents.**

Membrane CFTR currents were measured by whole cell patch clamp techniques. Cells on a cover slip were mounted in a chamber (volume ~400 µl) and perfused at 2~4 ml/min with an extracellular solution containing 150 mM *N*-methyl-D-glucamine-Cl, 1 mM $MgCl_2$, 1 mM $CaCl_2$ and 10 mM Hepes-Tris at pH 7.4. The osmolarity of the standard solution was 300±10 as measured by a vapor pressure osmometer (Advanced Micro Osmometer, MA, USA). The internal pipette solution was composed of 150 mM *N*-methyl-D-glucamine-Cl, 1 mM $MgCl_2$, 1 mM EGTA, 0.5 mM ATP and 10 mM HEPES at pH 7.3. The $[Ca2+]_i$ was buffered in the presence of 10 mM BAPTA to 1 nM or to other intracellular concentrations as indicated. Patch pipettes were pulled from Corning 7052 glass and had a resistance of 5-7MΩ. Recordings were made with an Axopatch 200B amplifier (Axon Instruments, Foster City, CA), and were digitized (1 kHz) for storage on a computer and analyzed using pCLAMP version 11.0.3 programs (Axon Instruments,

Burlingame, CA) as previously described.(12) The voltage protocol applied a holding potential –40 mV with a ramp from -100 mV to +100mv over 450 ms at 2 second intervals (for real-time tracings). The step protocol used a holding potential -40mV, with steps from -100mV to +100mV over 450ms in 20mV increments. Pipette voltages (Vp) correspond to the membrane potential, and upward deflections of the current trace indicate outward membrane current. Results are compared with control studies measured on the same day to minimize any effects of day-to-day variability and reported as current density (pA/pF) to normalize for differences in cell size. Capacitance and access resistance were monitored continuously.

**Measurement of transmembrane member 16A protein (TMEM16A) Cl- currents.**

Cells on a coverslip were mounted in the chamber, and whole cell currents were measured during basal and perfused conditions with a standard extracellular solution containing (in mM) 140 NaCl, 4 KCl, 1 CaCl2, 2 MgCl2, 1 KH2PO4, 10 glucose, and 10 HEPES/NaOH (pH 7.40). The standard intracellular (pipette) solution for whole cell recordings contained 130 mM KCl, 10 mM NaCl, 2 mM MgCl2, 10 mM HEPES/KOH, 0.5 mM CaCl2, and 1 mM EGTA (pH 7.3), corresponding to a free Ca2+ concentration of 100 nM. Patch pipettes, which were pulled from Corning 7052 glass, had a resistance of 4–6M. Recordings were made with an Axopatch 200B amplifier (Axon Instruments, Foster City, CA), digitized (2 kHz), and analyzed using pCLAMP version 11.0.3 (Axon Instruments, Burlingame, CA), as previously described.(13) Two voltage protocols were utilized: 1) holding potential of -40 mV with 450 ramps from -100 mV to +100 mV at 2-s intervals and 2) holding potential of -40 mV with 450-ms steps from -100 to +100 mV in

20mV increments. Current-voltage relations were generated from the “step” protocol. Results are compared with control studies measured on the same day to minimize effects of day-to-day variability and reported as current density (pA/pF) to normalize for differences in cell size.(12)

**Measurement of volume-activated Cl- currents.**

Coverslips with NMC cells were transferred to the recording chamber and perfused with a standard external solution with the following composition: 145 mM NaCl, 1 mM CaCl2, 1 mM MgCl2, 10 mM HEPES, and 10 mM D-glucose, pH 7.4 (NaOH). Osmolarity was adjusted with mannitol to 310 ± 5 mOsm using the Advanced Micro Osmometer (Model 3300, Advanced Instruments Inc.). For measuring swelling-activated currents and volume changes, an isotonic solution containing 85 mM NaCl, 1 mM CaCl2, 1 mM MgCl2, 10 mM HEPES, 120 mM D-mannitol, and 10 mM D-glucose, pH 7.4, with NaOH (310 ± 5 mOsm) was used and cell swelling was induced by omitting mannitol from the solution to reach osmolarity of 190 mOsm for most volume-activated studies.(14) Patch-clamp experiments were performed in the standard whole-cell configuration at room temperature (22°C–25°C) using an Axopatch 200B amplifier (Axon Instruments). Patch pipettes had resistances between 4 and 6 mΩ after filling with the standard intracellular solution that contained the following: 90 mM cesium (Cs)-chloride, 50 mM Cs-aspartate, 1 mM Mg-ATP, 10 mM HEPES, and 2 mM EGTA, pH 7.3 (cesium hydroxide). Digidata −1440A and pClamp 13 software (Molecular Devices) were used for data acquisition and analysis. The current was recorded by 450-ms rapid alteration of membrane potential from −100 to +100 mV every 2 s from a holding potential of -40 mV. The

current recorded at +100 mV was used to calculate the maximum current density (pA/pF). To observe the voltage and time dependency of the current profile, step pulses were applied from a holding potential of -40 mV to test potentials of −100 to +100 mV in +20 mV increments. The current was filtered at 1 kHz and sampled at 10 kHz. Results are compared with control studies measured on the same day to minimize any effects of day-to-day variability and reported as current density (pA/pF) to normalize for differences in cell size. Data are presented as mean ± SE. Origin 2018 (OriginLab) was used for data analysis and display.

**Ca2+ imaging.**

Cells were cultured for 24h on 10 mm glass coverslips and then loaded with 2.5 μg/ml of fura-2 AM (TEF Laboratories, Austin, TX, USA) in isotonic extracellular buffer containing: 140 mM NaCl, 4 mM KCl, 2 mM $CaCl_2$, 1 mM $MgCl_2$, 1 mM $KH_2PO_4$, 10 mM glucose, 10 mM HEPES (pH 7.4) supplemented with 0.01% pluronic F127 for 30 min at 37°C. The coverslip was placed in the perfusion chamber on the stage of an inverted fluorescent microscope (Nikon TE2000). Changes of $[Ca2+]_i$ were measured at excitation wavelength of 340 nm for calcium-bound fura-2 AM and 380 nm for calcium-free fura-2 AM, emission wavelength of 510 nm. Experiments were performed at room temperature. Ca2+ studies were conducted on 20 ~30 cells per field and repeated in three technical replicates.

**Measurement of ATP release.**

Cellular ATP release was studied using the luciferin-luciferase (L-L) assay as previously described.(15, 16) Cells on 35 mm tissue-culture-treated dishes (Falcon, Becton Dickinson Labware, Franklin Lakes, NJ) were washed with PBS (600 µl x 2), 600 µl Optimem (Gibco) containing L-L (Fl-ATP Assay Mix, Sigma-Aldrich, St. Louis, MO) added, and then placed into a modified Turner TD 20/20 Luminometer. After a 10-minute equilibration period, a basal reading was obtained and then an equal volume of isotonic buffer (200 µl) was added (to account for ATP release due to mechanical stimulation) followed by addition of hypotonic buffer. Readings were performed as cumulative bioluminescence over a 15 second interval and quantified as arbitrary light units (ALU's). Standard calibration curves were performed with known amounts of ATP added to the L-L Optimem reagent during cell-free conditions. All luminescence values are reported as relative change from basal luminescence per total protein level in the sample (measured in µg per ml) to control for any potential differences in luciferase activity or confluency between samples, respectively.(17)

**Immunofluorescence**

Localization of CFTR, TMEM16A, CK19, and LRRC8A was performed in confluent NMC monolayers. NMC on collagen coated tissue culture chamber slides (BD BioCoat) were fixed in 25% acetic acid/75% ethanol (v/v) for 10 min or 4% paraformaldehyde permeabilized with 1% Triton-X 100 for 10 min, incubated with 5% normal donkey serum, and then incubated overnight at 4°C with either rabbit anti-CFTR antibody (ACC-034, Alomone Labs, Jerusalem, Israel 1:200), anti-TMEM16A (ABN1669, Chemicon, 1:200), anti-CK19 (10712-1-AP, Proteintech, 1:200), or anti-LRRC8A (ACC-001, Alomone Labs,

1:200), followed by Dylight 488 conjugated donkey anti-rabbit (Jackson Immunoresearch, 1:600) and counter-labeled with DAPI (Advanced Cell Diagnostics) to visualize nuclei. Control cells were prepared by omitting either primary or secondary antibodies from the incubation solution. The slides were cover slipped with Mowiol Æ 4-88 with 2.5% DABCO and left overnight in the dark at room temperature. The slides were imaged using the Leica TCS SP5 confocal microscope (Leica Micro-systems, CMS GMBH) with custom software (Leica Micro-systems LAS AF). Images were acquired using a frame size of 512 x 512 pixels and 4-line averaging was used to remove noise from the image. Images were then imported in ImageJ (http://rsb.info.nih.gov) using the LOCI Bio-formats plug-in (University of Wisconsin, Madison).

**Total RNA isolation and RT-PCR analysis.**

RNA was extracted from the cells using Trizol (Invitrogen) followed by a DNase I treatment using the RNA Clean and Concentrator Kit (Zymo Research, catalog no. R1013). The DNase I-treated RNA (1 µg) was converted to complementary DNA (cDNA) using iScript Reverse Transcription Supermix (Bio-Rad, catalog no. 1708840) per the manufacturer's instructions. The cDNA was diluted 1:4 with water. PCR products representing the marker paralogs were detected with a 20 µl PCR reaction mixture containing sequence-specific primers (Table 1), GoTaq Master Mix (Promega, catalog no. M7132), and a 1/20 volume of cDNA. The mixture was subjected to 35 cycles of amplification (denaturation at 95°C for 30 s, annealing at 56°C for 30 s, and extension at 72°C for 20 s). The amplified DNA was resolved on a 2% agarose gel alongside a 50 bp ladder.

**Table 1: List of primers used for quantitative real-time PCR analysis.**

| | | | | |
|---|---|---|---|---|
| Alpha fetoprotein | *Afp* | *Mus musculus* | Forward: GATAGCTTCCACGTTAGATTCCT Reverse: GTCATTTTGTTCACTTCCTCCTC | 117 |
| Albumin | *Alb* | *Mus musculus* | Forward: GAAGTGCTCCAGTATGCAGAA Reverse: ACTTTGGTCAGGTCTGTTGC | 129 |
| Anoctamin 1, calcium activated chloride channel | *Ano1 (Tmem16a)* | *Mus musculus* | Forward: AAGTTTGTCACCGAGCTACG Reverse: GATGAAGTCAGACGTGAAGGAG | 138 |
| Collagen, type I, alpha 1 | *Col1a1* | *Mus musculus* | Forward: CGCAAAGAGTCTACATGTCTAGG Reverse: CATTGTGTATGCAGCTGACTTC | 136 |
| Cystic fibrosis transmembrane conductance regulator | *Cftr* | *Mus musculus* | Forward: GTCAAAGCTTGCCAACTACAG Reverse: GCTCTTGCTAAAGAAATCCTTGC | 122 |
| Epithelial cell adhesion molecule | *Epcam* | *Mus musculus* | Forward: TGGTGTCATTAGCAGTCATCG Reverse: GGCATTAAGCTCTCTGTGGAT | 125 |
| Gamma-glutamyl transferase | *Ggt1* | *Mus musculus* | Forward: CTGACGTATCACCGTATCGTG Reverse: AGAACTCAGAGCTCATGTTGC | 118 |
| Hepatocyte nuclear factor-1-beta | Hnf1b | *Mus musculus* | Forward: CACCTCTCTCAACACCTCAAC Reverse: CTGGACTGTCTGGTTGAACTG | 120 |
| Hepatocyte nuclear factor 4 alpha | Hnf4a | *Mus musculus* | Forward: TCCAGTTCATCAAGCTCTTCG Reverse: TGTTCTTGCATCAGGTGAGG | 127 |
| Keratin 7 | *Krt7* | *Mus musculus* | Forward: CTGAGAATGAGTTTGTGTTGCTG Reverse: TGAAGGGTCTTGAGGAAGTTG | 119 |

| | | | | |
|---|---|---|---|---|
| Keratin 19 | *Krt19* | *Mus musculus* | Forward: CTCCCGAGATTACAACCACTAC Reverse: CGAGCATTGTCAATCTGTAGGA | 108 |
| Leucine-rich repeat-containing protein 8A | *Lrrc8a* | *Mus musculus* | Forward: CACAACAACCTGACCTTCCTC Reverse: CCGTAGCTTCCGACACTG | 123 |
| SRY-Box Transcription Factor 9 | *Sox9* | *Mus musculus* | Forward: CGACCCATGAACGCCTT Reverse: GTCTCTTCTCGCTCTCGTTC | 139 |
| TATA box binding protein | *Tbp* | *Mus musculus* | Forward: TGTATCTACCGTGAATCTTGGC Reverse: CCAGAACTGAAAATCAACGCAG | 148 |
| Transient receptor potential cation channel subfamily V Member 4 | *Trpv4* | *Mus musculus* | Forward: CTGGAGATCCTGGTGTACAAC Reverse: GACCACGTTGATGTAGAAGGAC | 130 |

**Western Blot.**

Total protein extracts were prepared from mouse cholangiocytes as follows. Cells were washed twice with ice-cold PBS and lysed at 4 °C with RIPA buffer (Thermo scientific, catalog no. 89900). Protein fractions were subjected to 7.5% SDS–polyacrylamide gel and transferred to nitrocellulose membranes. After blocking, immunoblots were incubated overnight with anti-mouse CFTR, 1:1000 (CFF, CFTR217). This was followed by incubation with peroxidase-conjugated goat antirabbit antibody (1: 10,000 dilution, Jackson Immuno Research Laboratories, Inc.) and visualized by the ECL$^{+}$ detection kit (GE Healthcare, England). Primary antibodies used were CK19 (10712-1-AP, Proteintech, 1:500), CK7 (SC-23876, Santa Cruz, 1:500), CFTR (Ab-737) (8B0860, Syd labs, 1:200), and TMEM16A/ ANO1(H-41) (SC-135235, Santa Cruz, 1:500).

**Statistics**.

Results are presented as the mean ± standard error, with *n* representing the number of culture plates or repetitions for each assay as indicated. Statistical analysis included Fisher's paired and unpaired t-test and ANOVA for multiple comparisons to assess statistical significance as indicated, and p values $< 0.05$ were considered to be statistically significant.

## RESULTS

### Modified culture conditions allow cholangiocyte differentiation and long-term expansion.

To determine the conditions that would support long-term growth and differentiation of cholangiocytes in culture, we isolated cholangiocytes from C57BL/6 mice and cultured them in media that consisted of 50% H69 medium and 50% conditioned medium (CM) obtained from 3T3-J2 cells cultured in H69 media for 3 days. The media was subsequently supplemented with ROCK inhibitor Y-27632. The isolation and culturing method described in the Methods section were used to create successful cell lines from 5 WT mice and 2 CFTR knockout mice. For consistency, we only used one cell line to obtain the morphological and functional data presented in this report.

Although the cells grew slowly for several days, after 7 days in culture with CM many colonies of epithelial cells were visible, with one cell colony giving rise to hundreds of cells (Figure 1A). In parallel, cells were cultured in non-CM medium (H69 medium only without 3T3 conditioning or ROCK inhibitor). These cells grew much slower, with doubling time greater than one week. Additionally, these cells acquired a squamous morphology, in contrast to cells grown in CM, which retained an epithelial cell morphology and eventually formed a confluent monolayer.

In the first week in culture, cells growing on plastic showed signs of senescence and had begun to form islands of spreading cells. This could be reversed by stimulation with CM, which induced differentiation and proliferation. Even at high splitting ratios, the cells

attained confluency within 7 days (Figure 1B). However, if CM were removed, cell growth slowed. This is demonstrated in Figure 1E, which shows that cell growth in CM was comparable to non-CM in the beginning but began to diverge after 7 days (upper panel). Trans epithelial resistance (TER), a measure of the strength of tight junctions,(18) is increased in CM compared to non-CM. If CM is removed after 13 days, the cell growth again diverges (lower panel), demonstrating that CM media facilitates cell growth and stronger cell-cell tight junctions.

Next, we assessed the replicative capacity of these cells over time under different conditions. NMC at passage 7 and passage 52 showed similar seeding efficiencies after subculture but grew at significantly different rates over a period of 4 weeks. In passage 7, the cells without CM grew much slower than the culture with CM in the first two weeks. However, the cell numbers rapidly caught up and were equivalent in both CM and non-CM by the third and fourth week (Figure 1C). This implies that if cells are allowed to differentiate in the early passages, they can grow indefinitely. However, after 52 passages, cells cultured in non-CM did not catch up to the number of cells with CM even after 4 weeks (Figure 1C). Light microscopy shows that cultured NMC were composed of uniformly sized, tightly packed, polygonal cells with closely apposed intercellular membranes. Within 2 weeks, the initial colonies had enlarged to form confluent monolayers composed of cells with a uniform morphology. At later passages, however, without CM the cultures began to display increasing heterogeneity in size, degree of spreading, and surface granularity (Figure 1D). Using CM, we have established 2 long-term NMC cultures, designated NMC-a and NMC-b according to the

sequence in which they were initiated. The NMC-a line has been passaged 50 times and maintained in vitro for more than 14 months. Together, these results show that CM stimulates NMC proliferation over time, and that these cells maintain their morphology and growth advantage compared to cells grown in non-CM.

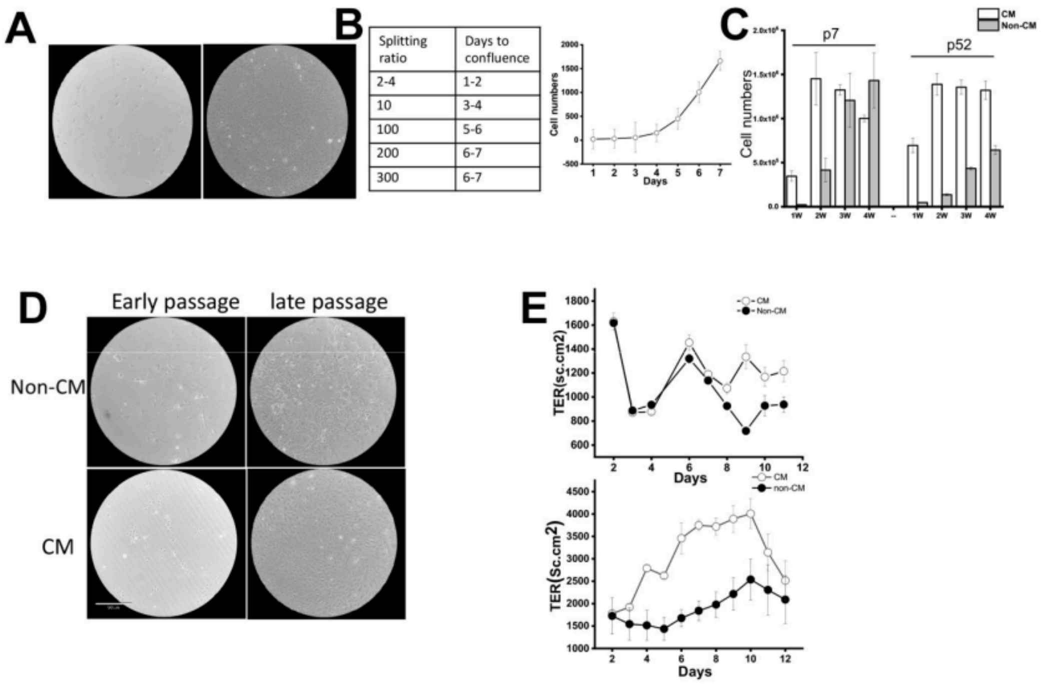


**Figure 1. Characterization of normal mouse cholangiocyte (NMC) in culture.** (A) Brightfield images of NMC cells treated with conditioned medium (CM) and non-CM. (B) Cell growth table (splitting ratio) and cell growth curve for NMC in CM; n=3 biological replicates. (C) Quantification of cell number in early vs late passage and CM vs non-CM; n=3 biological replicates per group. (D) Brightfield images of NMC cells in early vs late passage and CM vs non-CM. (E) Measurement of trans-epithelial resistance (TER)

in NMC grown in CM vs non-CM (upper panel), and after removal of CM at day 13 (lower panel); n=4 biological replicates per group.

**Normal mouse cholangiocytes (NMC) express and retain markers of differentiated biliary epithelium over time.**

Cholangiocyte genes *Epcam*, *Ggt1*, *Hnf1β*, *Krt7*, *Krt19*, *Sox9* were expressed in both early and late passages but were absent in hepatocyte cultures (Figure 2A). In contrast, cholangiocytes do not express hepatocyte markers *Alb*, *Afp*, and *Hnf4a*, or fibroblast marker *Col1a1* (Figure 2B). We next examined the expression of the Cl- channel CFTR, which is restricted to cholangiocytes, as well as the Cl- channels LRRC8A and TMEM16A and the Ca2+ channel TRPV4, which are expressed in cholangiocytes as well as other cell types. As shown in Figure 2C, NMC expressed all three different Cl- channels as well as TRPV4. This finding was confirmed by immunostaining, which revealed CFTR, TMEM16A, and LRRC8A on the plasma membrane (Figure 2D). Removal of CM did not reduce expression of biliary marker CK19 (Figure 2E). Furthermore, Western blotting of whole-cell lysates from NMC harvested at various time points after removal of CM shows that expression of CK7, CK19, CFTR, and TMEM16A remains consistent over time (Figure 2F). Therefore, NMC express cholangiocyte markers after isolation when cultured with or without CM, as well as in long-term culture.

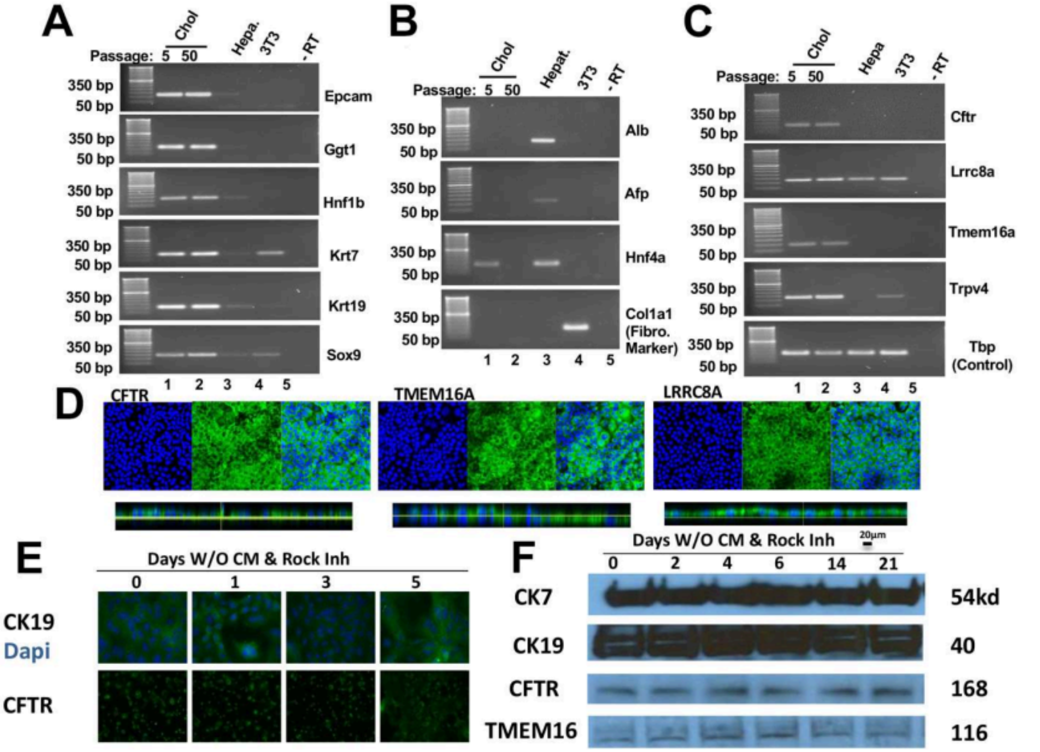


**Figure 2. Analysis of cholangiocyte marker expression in NMC.** (A) RT-PCR for cholangiocyte genes (*Epcam, Ggt1, Hnf1b, Krt7, Krt19, Sox9*) in NMC cultured for 5 or 50 passages, primary hepatocytes, and 3T3 cells. RT lane = no reverse transcriptase (negative control). (B) RT-PCR for hepatocyte and fibrosis genes (*Alb, Afp, Hnf4a, Col1a1*) in NMC cultured for 5 or 50 passages, primary hepatocytes, and 3T3 cells. (C) RT-PCR for cholangiocyte transporters (*Cftr, Lrrc8a, Tmem16a, Trpv4*) in NMC cultured for 5 or 50 passages, primary hepatocytes, and 3T3 cells. (D) Immunostaining for three Cl- channels (CFTR, TMEM16A, LRRC8A) on polarized NMC. (E) CK19 immunostaining on NMC cultured in CM and non-CM. (F) Comparison of cholangiocyte markers C7, CK19, CFTR, and TMEM16A in NMC cultured in CM and non-CM by

Western blot. One representative well was harvested for protein at the indicated time point to represent the effect of CM removal in the NMC cell line over time.

**NMC retain their biophysical properties and Cl- conductances in culture.**

To assess the retention of biophysical properties, electrophysiological techniques are commonly employed. 86 Patch-clamp recordings can be performed to measure ion currents across the cell membrane, including chloride currents. This allows the evaluation of the specific ion channel activities and the biophysical properties of the cholangiocytes in culture. Cells from the same line used for morphological and marker analysis were used for each of the patch clamp studies described below. Cells were analyzed from different plates on multiple dates, and measurements were repeated on at least three different days for reproducibility.

**A. CFTR Cl- currents**

44 Cystic fibrosis transmembrane conductance regulator (CFTR) is a Cl- channel expressed on the apical surface of cholangiocytes that is activated by cAMP/PKA.(1) Unlike other Cl- channels, CFTR is only expressed on cholangiocytes, not on all liver cells; thus, we sought to establish CFTR function as a primary indicator of successful long-term cholangiocyte culture. To assess the functionality of CFTR channels in NMC cells, 8 membrane currents were measured via whole cell patch-clamp techniques. Cells on a coverslip were mounted in a chamber and currents were measured with a standard CFTR extracellular solution (see Methods section). Cells were then exposed to 10uM forskolin+10uM isobutyl methylxanthine (IBMX), which induced current typical of CFTR

activation. As shown, the current-voltage relationship was linear, demonstrating that NMC cells exhibit cAMP-stimulated Cl-currents.

We then compared early passage (p7) and later passage (p50) NMC with and without CM (Figure 3A-B). The results show that there is no significant difference between early passage (n=9) and late passage(n=15) in forskolin cocktail-induced Cl- current. Additionally, there is no significant difference between the cultures with CM (n= 12) and without CM (n=11) (Figure3C). The average CFTR current value was between 10 to 20 pA/pF in all groups. We therefore concluded that the cells exhibit CFTR functionality regardless of the number of passages and despite the removal of CM.

To strengthen our findings, CFTR specific inhibitor 172 was used to verify the forskolin cocktail activated current.(18) When NMC cells were preincubated with 172 from passage 12 to passage 15, CFTR currents were remarkably inhibited (Figure 3E). NMC cells were transfected with siRNA against CFTR, which knocks down the mature, complex-glycosylated C band, leaving the core glycosylated form of the protein (B band) intact(19) (Figure 3D). No currents were detected in NMC after knockdown of CFTR (Figure 3E), confirming that the Cl- currents were a result of CFTR activation.

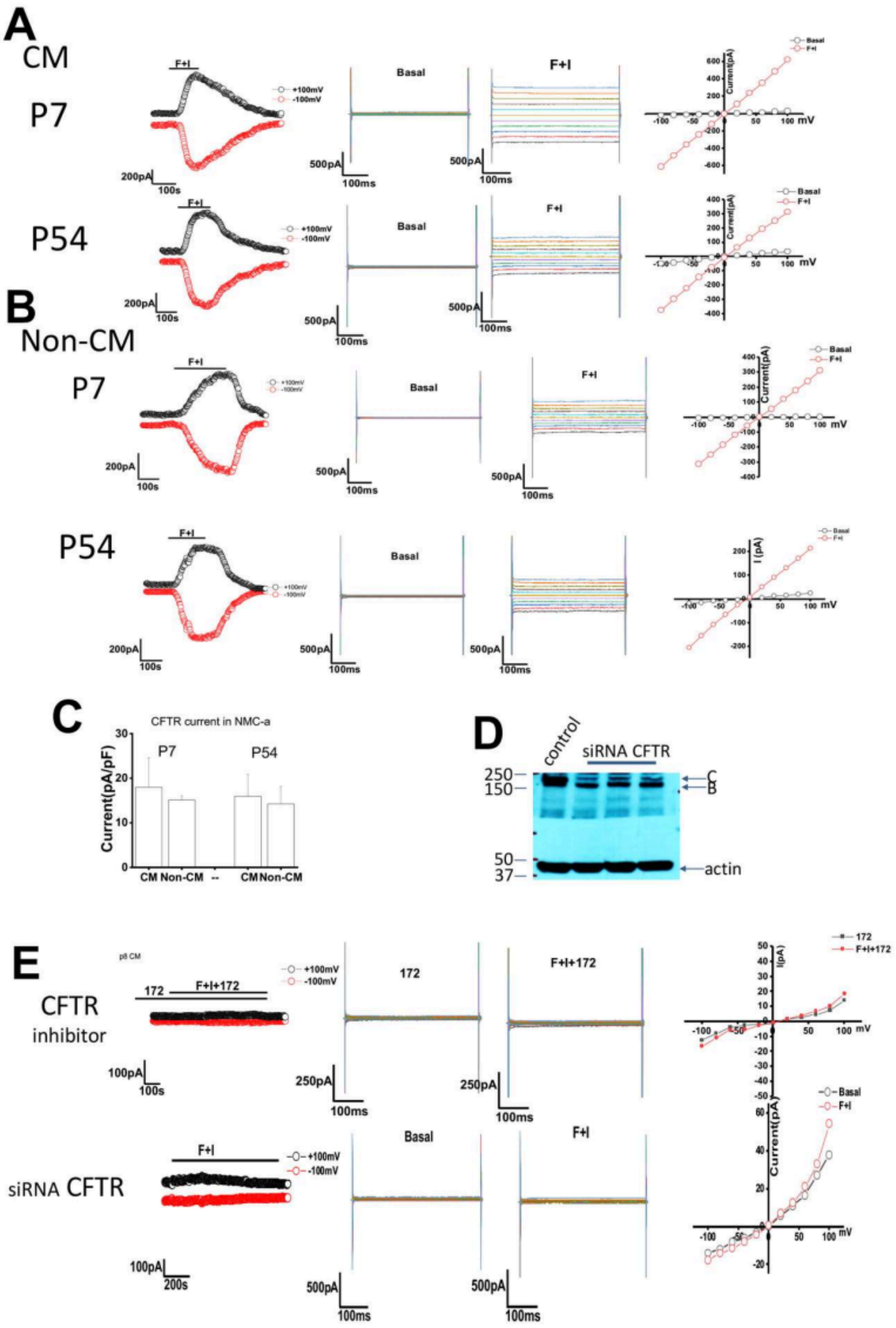
A
CM
P7
P54
F+I
+100mV
-100mV
200pA
100s
Basal
500pA
100ms
Current(pA)
mV
B
Non-CM
C
CFTR current in NMC-a
P7
P54
Current(pA/pF)
CM
Non-CM
D
control
siRNA CFTR
250
150
50
37
C
B
actin
E
CFTR
inhibitor
172
F+I+172
100pA
250pA
siRNA CFTR
200s

**Figure**

**3. Measurement of CFTR Cl- currents in NMC.** (A-B) Left: Whole cell patch clamp studies in NMC exposed to 100μM forskolin and 10μM IBMX. Currents measured at −100 mV (bottom red circles) and at +100 mV (top black circles) are shown. Middle: Currents were measured in NMC during basal (control) conditions and in response to forskolin and IBMX (F + I) using STEP protocol. Right: Current-voltage (I-V) plot generated by STEP protocol. (A) NMC cultured in CM. Top: NMC from passage 7; bottom: NMC from passage 54. (B) NMC cultured in non-CM. Top: NMC from passage 7; bottom: NMC from passage 54. (C) Summary of CFTR currents in early passage vs late passage and CM vs non-CM. Data reflects the total number of cells measured from three technical replicates. For CM p7 n=13 cell currents were measured; for non-CM p7 n=9; for CM p54 n=8; for non-CM p54 n=8. Comparison of multiple groups was performed by one-way ANOVA followed by Tukey;'s post hoc test. (D) Western blot shows successful knockdown of the CFTR C band after treating NMC with CFTR siRNA. (E) Whole cell patch clamp studies in NMC treated with either CFTR inhibitor 172 (top), or transfected with CFTR siRNA (bottom) and exposed to 100μM forskolin and 10μM IBMX.

**B. ATP-stimulated currents**

Transmembrane member 16A protein (TMEM16A) is a Ca2+-activated Cl- channel that is expressed on the cholangiocyte plasma membrane.(13) Expression of TMEM16A increases transepithelial secretion in response to extracellular nucleotides. To determine the functionality of TMEM16A in NMC, we measured whole-cell currents using patch clamping. Exposure of NMC cells to ATP resulted in instantaneous

activation of Cl- currents with time-dependent activation at positive potentials (greater than +60 mV), outward rectification, and reversal at 0 mV ($E_{Cl^-} = 0$) consistent with the properties of TMEM16A. In some cases, the ATP-stimulated currents demonstrated an oscillatory pattern. Thus, fresh isolated primary cholangiocytes express TMEM16A and exhibit Ca2+-activated Cl- currents in response to ATP. We did not observe any significant difference between current measured in early passage cells (p8) and late passage (p50) cells (Figure 4A, D). Additionally, culturing NMC in non-CM does not affect the function of TMEM16A (Figure 4B, D). Finally, ATP activated current was abolished by apyrase, which hydrolyzes ATP (Figure 4C), demonstrating the specificity of this response.

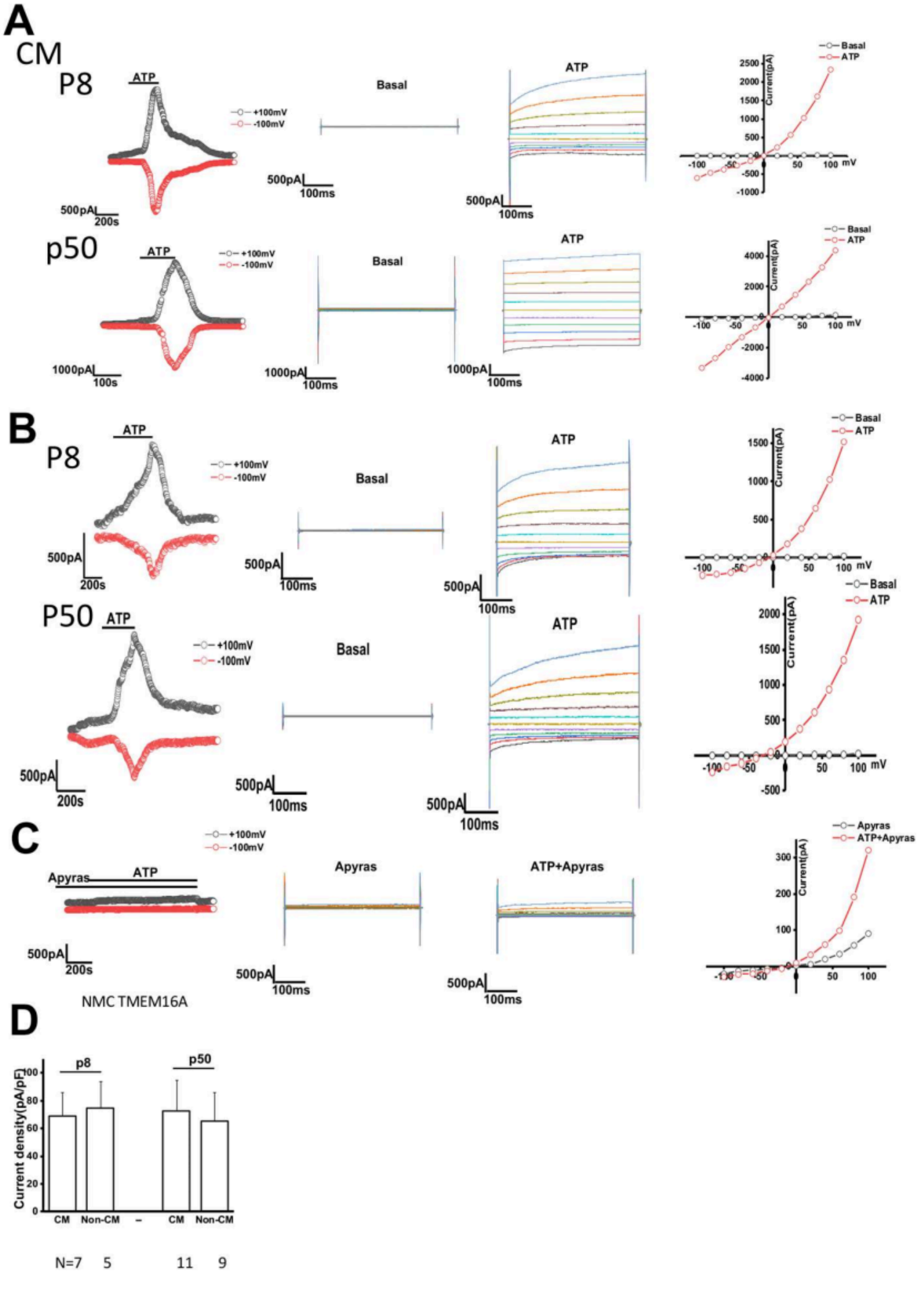
A
CM
P8
ATP
+100mV
-100mV
500pA
200s
Basal
500pA
100ms
ATP
500pA
100ms
Basal
ATP
Current(pA)
mV
p50
ATP
1000pA
100s
Basal
1000pA
100ms
ATP
1000pA
100ms
B
P8
ATP
500pA
200s
Basal
500pA
100ms
ATP
500pA
100ms
P50
ATP
500pA
200s
Basal
500pA
100ms
ATP
500pA
100ms
C
Apyras
ATP
500pA
200s
Apyras
500pA
100ms
ATP+Apyras
500pA
100ms
Apyras
ATP+Apyras
NMC TMEM16A
D
Current density(pA/pF)
p8
p50
CM
Non-CM
CM
Non-CM
N=7 5 11 9

**Figure 4. Measurement of the TMEM16A-mediated ATP activated Cl- current in NMC.** (A-C) Left: Whole cell patch clamp studies in NMC exposed to 100μM ATP. Currents measured at −100 mV (bottom red circles) and at +100 mV (top black circles) are shown. Middle: Currents were measured in NMC during basal (control) conditions and in response to ATP using STEP protocol. Right: Current-voltage (I-V) plot generated by STEP protocol. (A) NMC cultured in CM. Top: NMC from passage 8; bottom: NMC from passage 50. (B) NMC cultured in non-CM. Top: NMC from passage 8; bottom: NMC from passage 50. (C) NMC treated with apyrase, which hydrolyzes ATP. (D) Summary of TMEM16A currents in early passage vs late passage and CM vs non-CM. Data reflects the total number of cells measured from three technical replicates. For CM p8 n=7 cell currents were measured; for non-CM p8 n=5; for CM p50 n=11; for non-CM p50 n=9. Comparison of multiple groups was performed by one-way ANOVA followed by Tukey;'s post hoc test.

### C. Volume-stimulated Cl- currents

Volume-stimulated Cl- channels such as leucine rich repeat-containing 8 subunit A (LRRC8A) are present in all cells and are responsive to cell swelling and increased volume.(20) To determine how changes in cell volume affect these channels in NMC, the cells were exposed to a 33% hypotonic solution. Under basal conditions with standard intra- and extracellular buffers, $I_{Cl}$ was small (−0.8 ± 0.2 pA /pF). Exposure to 33% hypotonic solution resulted in activation of currents within 4 to 6 minutes, increasing current density to −14.0 ± 0.9 pA/pF at −80 mV. The currents measured at 0 mV were transient and of small magnitude, while the currents measured at −80 mV

were sustained for the duration of hypotonic exposure and were fully reversible within 10 min of isotonic solution. These large volume-stimulated currents exhibited reversal near -15 mV ($E_{Cl}$), outward rectification and time-dependent inactivation at depolarizing potentials above +60 mV, characteristics different from ATP-stimulated $Cl^-$ currents previously described in these cells (Figure 5A). The magnitude of volume-stimulated currents was 3-fold greater than the currents stimulated by cAMP (CFTR current). There was no significant difference between the cells cultured with CM or in non-CM. Similar results were also obtained from early passage and late passage NMC (Figure 5A, B and D). Finally, treatment with LRRC8A specific inhibitor DCPIB completely abolished current activity (Figure 5C). These results confirm that NMC respond to hypotonic conditions and produce volume-stimulated currents regardless of culturing conditions and number of passages.

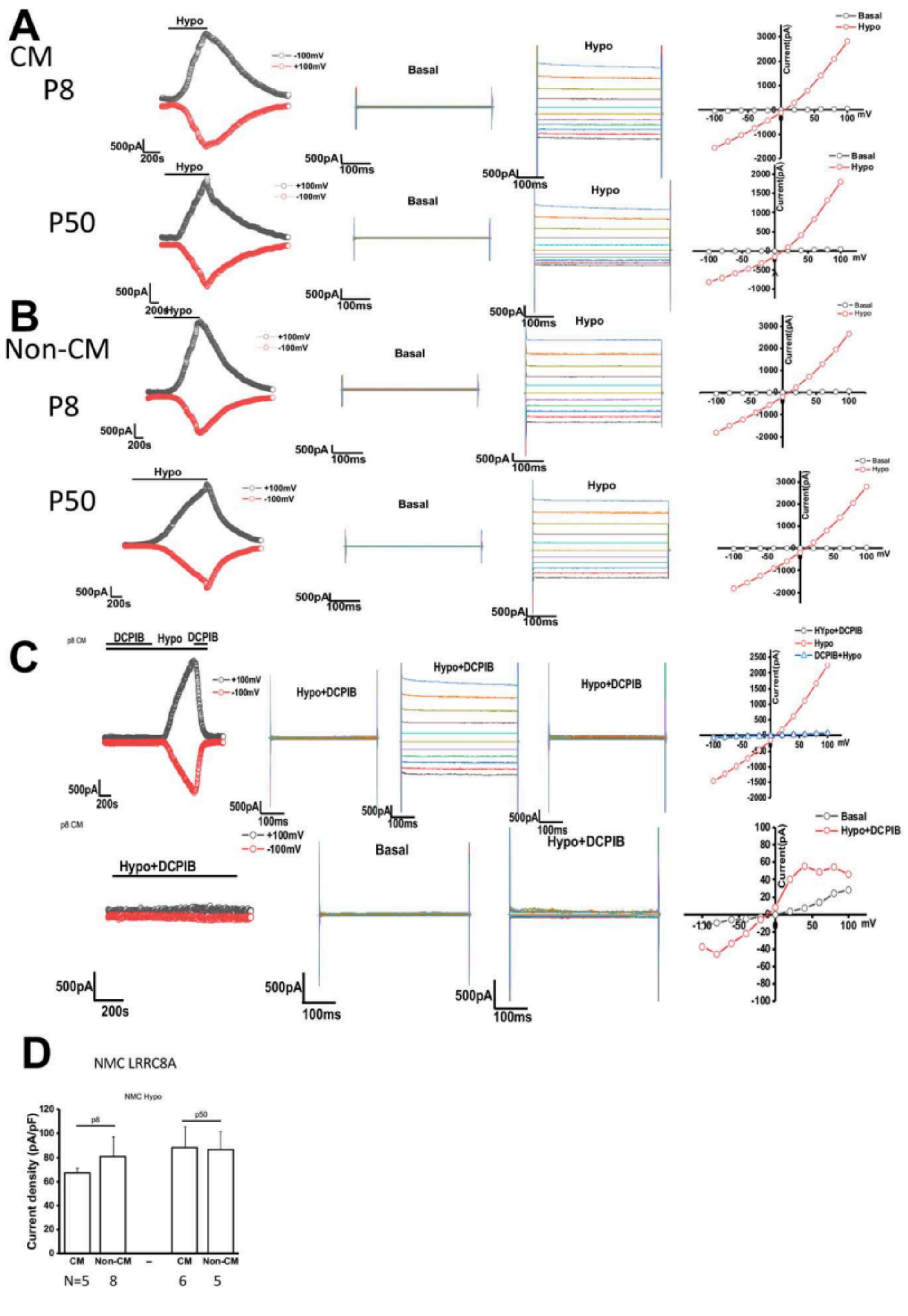

A
CM
P8
P50
B
Non-CM
C
D
Hypo
Basal
Hypo+DCPIB
DCPIB+Hypo
DCPIB
-100mV
+100mV
500pA
200s
100ms
Current(pA)
mV
NMC LRRC8A
NMC Hypo
Current density (pA/pF)
p8
p50
CM
Non-CM
N=5
8
6
5

**Figure 5. Measurement of the LRRC8A-mediated volume-stimulated Cl- current in NMC.** (A-C) Left: Whole cell patch clamp studies in NMC exposed to 33% hypotonic solution. Currents measured at −100 mV (bottom red circles) and at +100 mV (top black circles) are shown. Middle: Currents were measured in NMC during basal (control) conditions and in response to 33% hypotonic solution using STEP protocol. Right: Current-voltage (I-V) plot generated by STEP protocol. (A) NMC cultured in CM. Top: NMC from passage 8; bottom: NMC from passage 50. (B) NMC cultured in non-CM. Top: NMC from passage 8; bottom: NMC from passage 50. (C) NMC treated with DCPIB, an inhibitor specific for LRRC8A. (D) Summary of LRRC8A currents in early passage vs late passage and CM vs non-CM. Data reflects the total number of cells measured from three technical replicates. For CM p8 n=5 cell currents were measured; for non-CM p8 n=8; for CM p50 n=6; for non-CM p50 n=5. Comparison of multiple groups was performed by one-way ANOVA followed by Tukey;'s post hoc test.

**Cholangiocytes from CFTR knock out mouse (CFMC) lack CFTR current but otherwise display similar biophysical properties to NMC.**

To further validate our culture methods, we isolated cholangiocytes from CFTR knockout mice using the same methods used for NMC. We successfully established CFTR knockout cholangiocyte cell lines and tested the activity of the three different Cl- channel activities. As expected, CFTR currents were absent when CFMC cells were exposed to forskolin cocktail (Figure 6A). However, TMEM16A currents were maintained (Figure 6B), and LRRC8A currents were also recorded after application of hypotonic solution (Figure 6C). Therefore, while CFTR currents were abolished in

CFMC due to loss of CFTR protein, all other currents were present. These studies indicate that our protocol is capable of maintaining both normal and genetically mutated cholangiocytes in culture.

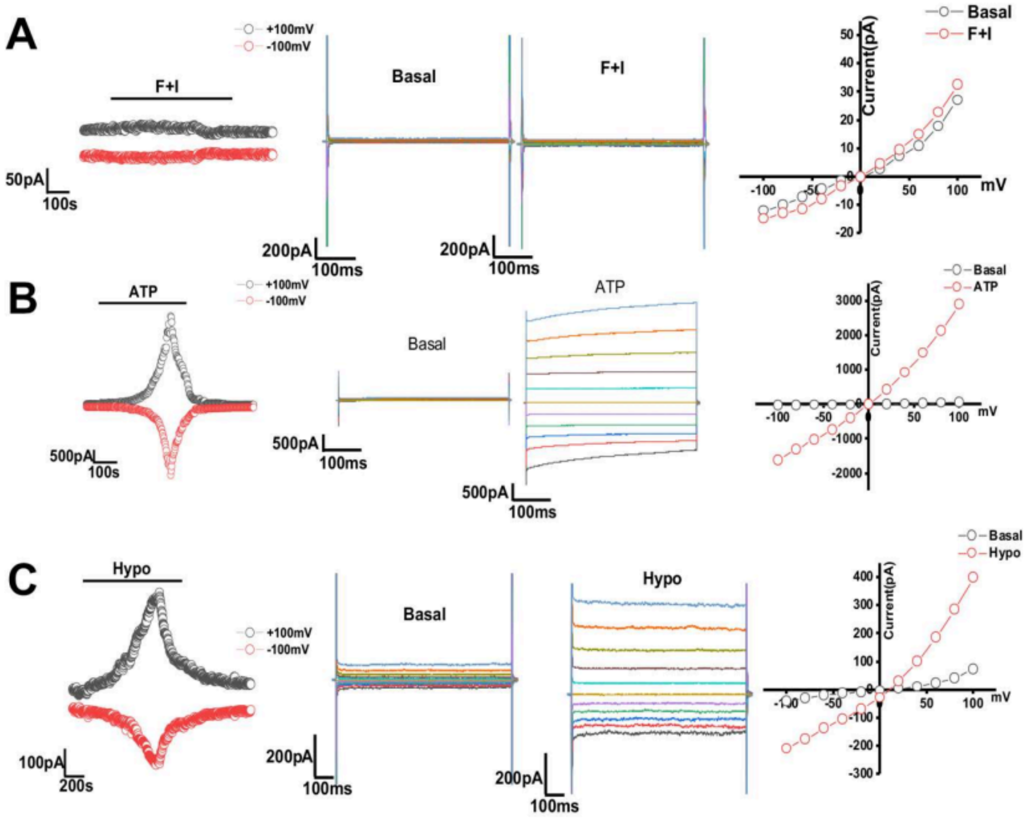


**Figure 6. Measurement of three different Cl- channels in cholangiocytes from CFTR knock out mouse (CFMC).** (A-C) Left: Whole cell patch clamp studies. Currents measured at −100 mV (bottom red circles) and at +100 mV (top black circles) are shown. Middle: Currents were measured in NMC during basal (control) conditions and in response to an activator (F + I, ATP, or hypotonic buffer) using STEP protocol. Right: Current-voltage (I-V) plot generated by STEP protocol. (A) CFMC exposed to 100μM

forskolin and 10µM IBMX. (B) CFMC exposed to 100µM ATP. (C) CFMC exposed to 33% hypotonic solution. Data are representative of measurements from three technical replicates.

**Both NMC and CFMC release ATP, resulting in increased intracellular Ca2+.**

To determine whether NMC cells release ATP in response to hypotonic solution, ATP in the extracellular media was detected using the luciferin-luciferase assay and quantified as arbitrary light units (ALU). Exposing the NMC to 33% hypotonic media resulted in a rapid increase in ATP release that occurred within seconds and was significantly increased over control cells exposed to isotonic exposure. Although the CFMC also responded to hypotonic solution, ATP release was decreased compared to the NMC (Figure 7A). 24-norUrsodeoxycholic acid (norUDCA), a synthetic analog of the hydrophilic bile acid UDCA, can induce bile acid elimination by stimulating Cl- channels. NorUDCA can also stimulate ATP release. Figure 7B shows that ATP release increased when cells were exposed to norUDCA. However, NMC cells released more ATP than norUDCA-treated CFMC. The decreased responsiveness to both hypotonic solution and norUDCA confirms CFTR's significant contribution to ATP release.

ATP release increases intracellular Ca2+, a key step in regulating ductular secretion and Cl- efflux.(21) Hypotonic solution and norUDCA increased intracellular Ca2+ in NMC, which was measured by fura-2 AM (Figure 7C). The specificity of this response was confirmed by treatment with apyrase, which hydrolyzes ATP and thus inhibits the increase of intracellular Ca2+. Parallel experiments with NMC and CFMC showed that

intracellular Ca2+ levels were blunted when CFMC were treated with norUDCA or hypotonic solution (Figure7C). Together, these studies demonstrate that functional CFTR in the cholangiocyte membrane is required for ATP release and accumulation of intracellular Ca2+.

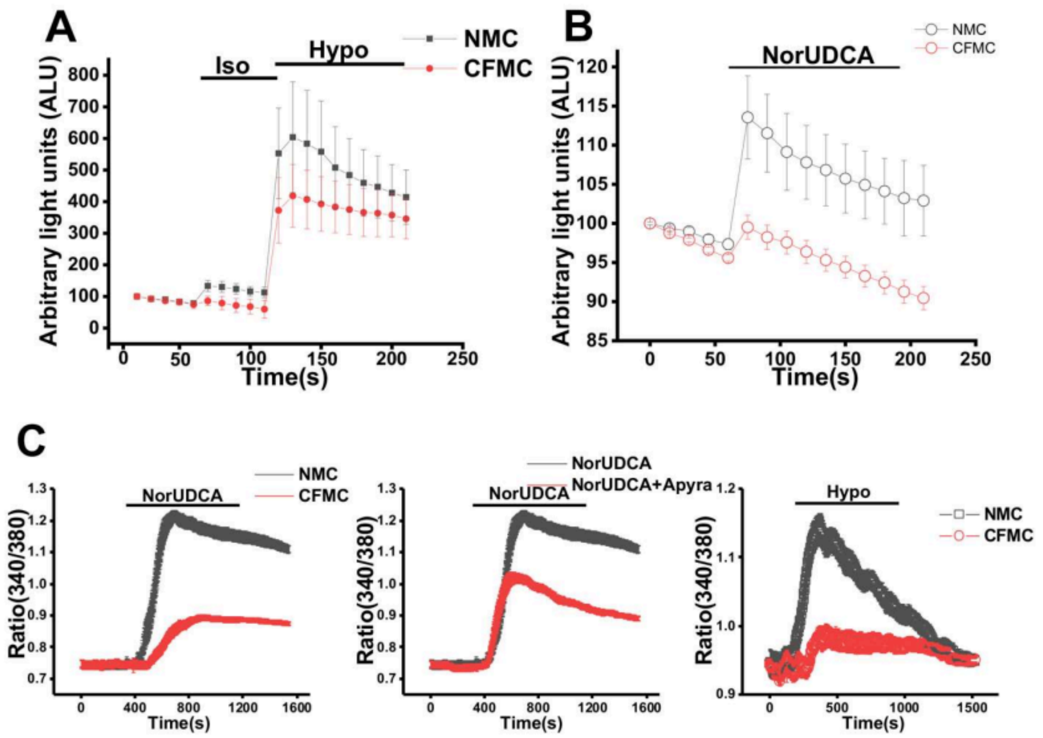


**Figure 7. Measurement of ATP release and changes in intracellular Ca2+ in NMC and CFMC.** (A) Hypotonic stress-induced ATP release from NMC (black circles) and CFMC (red circles). ATP in the extracellular media was detected using the luciferin-luciferase assay and quantified as arbitrary light units (ALU). The y-axis represents relative increase from basal luminescence (expressed as relative ALU/µg/ml protein) after addition of 33% hypotonic media. (B) NorUDCA-induced activation of TMEM16A channels in NMC (black circles) and CFMC (red circles). As in (A), ATP release is

quantified as ALU. (C) Intracellular Ca2+ changes induced by NorUDCA and 33% hypotonic media. Left: Comparison of Ca2+ accumulation in NMC (black) and CFMC (red) after treatment with NorUDCA. Middle: Comparison of Ca2+ accumulation in NMC (black) and NMC + ATP inhibitor apyrase (red) after treatment with NorUDCA. Right: Comparison of Ca2+ accumulation in NMC (black) and CFMC (red) after treatment with hypotonic media. Changes in intracellular calcium were plotted as a ratio of calcium-bound fura-2 AM (340 nm) and calcium-free fura-2 AM (380 nm). For A and B, n=3 biological replicates per group. For C, studies were conducted on 20 ~30 cells per field, and repeated in three technical replicates. Representative images are shown.

**DISCUSSION**

Cholangiocytes, which form the lining of the biliary tract, play an essential role in bile formation and secretion in the liver. These cells, which are heterogenous in structure and function, are polarized, possessing an apical and a basolateral plasma membrane. Under physiological conditions, cholangiocytes modulate bile in the ductal lumen through transport of Cl-, $HCO_3$-, and water across the apical membrane, which results in alkalinization of bile and increased bile volume.(1, 22) This process is driven by increases in cAMP and Ca2+. Cholangiocytes also contribute to bile modification through absorption of ions, bile acids, glucose, and other molecules. Notably, cholangiocytes are both a target of and a participant in various disease states where bile flow is impeded or decreased.(3) Despite their importance in both physiology and pathophysiology, the use of primary cholangiocytes for *in vitro* studies has been limited in part by difficulties in isolating these cells from the intrahepatic mass. Long-term culture of cholangiocytes from normal liver has also been hampered by the relatively small numbers of ductal cells obtained from isolation and by complex culture procedures.(8, 23) Thus, hyperplastic or immortalized cholangiocytes have been widely used as a surrogate.(5, 6) These cells have been well characterized; however, constitutively proliferating cells can acquire alterations in gene expression and phenotype over time that may affect their physiological function. To avoid these pitfalls, we have developed a protocol for establishing primary cultures of NMC from normal adult mouse liver that is both reproducible and allows for long-term maintenance of primary cholangiocytes in culture.

Previously, normal rat cholangiocytes (NRC) were shown to retain many of the phenotypic features of cholangiocytes *in situ* when cultured in the presence of collagen and defined media supplements, including the ability to form monolayers.(24) NRC have since been utilized in transport and electrophysiologic studies to characterize glucose transport, Na+-dependent bile acid transport, and cAMP-stimulated Cl- secretion.(25-28) By replicating these conditions in the preparation of our primary mouse cell lines, we were able to obtain similar findings and demonstrate the utility of NMC cells as a model for investigating the cellular mechanisms responsible for cholangiocyte secretion and bile formation.

To isolate cholangiocytes, we first precultured intact bile duct fragments in a collagen gel for seven days to reproduce the methodology used for isolating NRC.(29) This technique was thought to be superior to previous protocols that use elutriation and antibody-coated magnetic beads to isolate cholangiocytes from enzymatically dissociated biliary trees because cholangiocytes can survive and proliferate within the ducts due to maintenance of cell contacts in ductal fragments.(5, 29) We employed separation and purification of cholangiocytes with EpCAM antibody only after the preculturing phase was complete. The role of extracellular matrix in maintaining cell shape, proliferation, and differentiation of epithelial cells is well known.(30) Our results using collagen gels seemed to confirm this. However, we could also bypass this step by dicing bile ducts into small pieces and using trypsin and DNAse to obtain single cell suspensions. Results from preliminary studies suggest that the growth and efficiency of

bile duct epithelial cells was not significantly different if first preincubated in collagen gels or cultured directly after enzymatic digestion.

Y-27632 is a selective inhibitor of Rho kinase (ROCK), which is involved in a variety of cellular processes, including contraction, adhesion, migration, proliferation, and apoptosis through its regulation of actin cytoskeleton assembly and cell contraction.(31, 32) Y-27632 was initially shown to increase the cloning efficiency of human embryonic stem cells, enhancing their survival by preventing apoptosis.(33) Since then, Y-27632 has been used to regulate the differentiation and proliferation of multiple types of stem cells.(34, 35) Notably, Y-27632 also induced indefinite cell proliferation of primary human keratinocytes in culture.(36, 37) This process, which is referred to as conditional immortalization or conditional reprogramming, produces conditionally reprogrammed cells (CRC) without the need for a feeder layer. Supplementing media with Y-27632 enables unlimited, feeder-free expansion of porcine airway epithelial cells while maintaining an epithelial phenotype.(38) Another technique to extend replicative capacity is the treatment of ROCK inhibitor in combination with conditioned medium. Medium that has been conditioned by exposure to fibroblasts such as the 3T3-J2 cell line used in our experiments contains secreted paracrine factors that can maintain cell viability and function.(39) The combination of Y-27632 and feeder cells has been successfully used to convert multiple epithelial cell types to a proliferative state while simultaneously maintaining a normal cellular phenotype.(40) Bone marrow mesenchymal stem cell differentiation into keratinocytes was also achieved when these cells were exposed to Y-27632 and keratinocyte CM.(41) In our study, primary NMC

had not undergone senescence even after 50 passages in the presence of both Y-27632 and CM, demonstrating the efficacy of combining a proliferative enhancer with stromal-derived paracrine signals that maintain physiological function.

Phenotypically, NMC display a phenotype consistent with a biliary origin, with a small diameter and an epithelial morphology. The colony formation assay also demonstrated that both Y-27632 and CM are required for optimal growth of the largest and most numerous cholangiocytes, clones with normal epithelial morphology. Under these culture conditions, cholangiocytes maintained expression of differentiated biliary markers after 52 passages and 14 months in culture. The retention of biophysical properties in cultured cholangiocytes can be influenced by the duration of culture, passage number, and culture environment. However, in NMC, the presence of these channels and the subsequent confirmation of their function by electrophysiological techniques indicate that these factors were not impacted by long-term culture. Importantly, when cultured in non-CM, NMC retain many of the same differentiation markers and functions as cells cultured in CM, albeit with slower growth kinetics. Although NMC need CM in early passages for expansion, once a cell line has been established, it can be maintained in non-CM, which reduces the time, effort, and costs associated with standard conditional reprogramming protocols.

The goal of this study was to determine isolation and culture conditions that not only extend in vitro life span but preserve differentiation capacity, including CFTR function. Human bronchial epithelial basal cells (HBECs) gradually lose CFTR function over

time.(42) Modifying the standard CRC protocol allowed for the long-term growth of both normal and cystic fibrosis (CF) HBECs while maintaining their capacity for differentiation.(11) Similarly, we were able to extend the life span of CF and non-CF bile duct epithelial cells and maintain primary-like cell characteristics, including multipotent differentiation potential and CFTR expression. We also demonstrated experimentally that NMC conduct $Ca^{2+}$ activated and volume-stimulated $Cl^-$ membrane currents, and that other physiological functions, such as ATP release and accumulation of intracellular $Ca^{2+}$, are preserved as well. We thus conclude that these cells more accurately reflect *in situ* cholangiocytes than other cells previously used to study CF that were altered by expression of viral oncogenes or telomerase.

There are important limitations to the current study. First, we utilized the same cell line for all functional analyses in order to maintain consistency. However, performing all analyses in NMC from a single animal does not allow for the assessment of biological variability and raises the issue of pseudoreplication, since the experimental replicates are technical repetitions of the same culture. Future studies utilizing additional animals and strains will be important to demonstrate reproducibility of the protocol. Second, the seeding density for the NMC cultures was low, ranging from one cell to several hundred. This is partly attributable to low yields, since cholangiocytes represent only 3% to 5% of the total cell mass.(3) The numbers were further reduced after purification with antibody-conjugated beads in order to get a highly homogeneous population of cholangiocytes. Nonetheless, despite their relative scarcity, primary cholangiocytes grew to confluence and maintained their proliferative capacity during long-term culture.

Additionally, we were able to successfully produce colonies from single cells and then expand these colonies to create cell lines, demonstrating clonal ability. Future studies will be needed to determine whether these NMC cell lines undergo genetic drift and/or chromosomal instability, and whether these cells ultimately undergo senescence in the absence of immortalization. The impact of this work would also be enhanced by successfully isolating and culturing human cholangiocytes using these techniques.

Despite multiple studies characterizing the phenotype of cholangiocytes in culture, maintaining these cells in a differentiated state over multiple passages has remained a challenge.(23) These limitations have prompted a search for alternative cell sources that are suitable for genetic and functional studies. Previous studies have shown that mouse cholangiocytes isolated from normal mice (BALB/c) and immortalized by transfection with the SV40 large-T antigen display features of freshly isolated small and large mouse cholangiocytes.(6, 43) Cholangiocytes can also be differentiated from induced pluripotent stem cells into cholangiocyte-like cells with gene expression profiles and marker expression similar to primary human cells.(7, 8) Organoids generated from bile ducts retain their original tissue characteristics and can be stably passaged.(9) Cholangiocytes isolated from diseased tissue may also provide a source of cholangiocytes to study the pathogenesis of cholangiopathies.(44) However, most of these studies lack a detailed assessment of transmembrane receptor functionality. We have used specialized isolation and culture techniques to maintain primary mouse cholangiocytes that not only retain biophysical properties and distinct Cl- conductances but also undergo an extended number of passages. The NMC produced from this

reliable and reproducible protocol should provide a vital tool that will advance our understanding of cholangiocyte physiology and pathobiology.

**Abbreviations:** Rho-associated protein kinase (ROCK); normal mouse cholangiocytes (NMC); chloride (Cl-); Epithelial Cell Adhesion Molecule (EpCAM); Calcium (Ca2+); conditioned medium (CM); trans-epithelial resistance (TER); cystic fibrosis transmembrane conductance regulator (CFTR); transmembrane member 16A protein (TMEM16A); leucine rich repeat-containing 8 subunit A (LRRC8A); 24-norUrsodeoxycholic acid (norUDCA); Ursodeoxycholic acid (UDCA)

**Acknowledgements:** The authors would like to acknowledge Dr. Andrew Feranchak for his many intellectual contributions to the field of cholangiocyte physiology. Graphical abstract was prepared using Biorender.com.

## REFERENCES


1. **Tabibian JH, Masyuk AI, Masyuk TV, O'Hara SP, and LaRusso NF**. Physiology of cholangiocytes. *Compr Physiol* 3: 541-565, 2013.
2. **Jalan-Sakrikar N, Guicciardi ME, O'Hara SP, Azad A, LaRusso NF, Gores GJ, and Huebert RC**. Central role for cholangiocyte pathobiology in cholestatic liver diseases. *Hepatology* 82: 834-854, 2025.
3. **Banales JM, Huebert RC, Karlsen T, Strazzabosco M, LaRusso NF, and Gores GJ**. Cholangiocyte pathobiology. *Nat Rev Gastroenterol Hepatol* 16: 269-281, 2019.
4. **Mano Y, Ishii M, Kisara N, Kobayashi Y, Ueno Y, Kobayashi K, Hamada H, and Toyota T**. Duct formation by immortalized mouse cholangiocytes: an in vitro model for cholangiopathies. *Lab Invest* 78: 1467-1468, 1998.
5. **Sirica AE, Mathis GA, Sano N, and Elmore LW**. Isolation, culture, and transplantation of intrahepatic biliary epithelial cells and oval cells. *Pathobiology* 58: 44-64, 1990.
6. **Ueno Y, Alpini G, Yahagi K, Kanno N, Moritoki Y, Fukushima K, Glaser S, LeSage G, and Shimosegawa T**. Evaluation of differential gene expression by microarray analysis in small and large cholangiocytes isolated from normal mice. *Liver Int* 23: 449-459, 2003.
7. **Florentino RM, Li Q, Coard MC, Haep N, Motomura T, Diaz-Aragon R, Faccioli LAP, Amirneni S, Kocas-Kilicarslan ZN, Ostrowska A, Squires JE, Feranchak AP, and Soto-Gutierrez A**. Transmembrane channel activity in human hepatocytes and cholangiocytes derived from induced pluripotent stem cells. *Hepatol Commun* 6: 1561-1573, 2022.
8. **Luce E, and Dubart-Kupperschmitt A**. Pluripotent stem cell-derived cholangiocytes and cholangiocyte organoids. *Methods Cell Biol* 159: 69-93, 2020.
9. **Chen W, Yao Q, Wang R, Fen B, Chen J, Xu Y, Yu J, Li L, and Cao H**. Highly Efficient Methods to Culture Mouse Cholangiocytes and Small Intestine Organoids. *Front Microbiol* 13: 907901, 2022.
10. **Sampaziotis F, Muraro D, Tysoe OC, Sawiak S, Beach TE, Godfrey EM, Upponi SS, Brevini T, Wesley BT, Garcia-Bernardo J, Mahbubani K, Canu G, Gieseck R, 3rd, Berntsen NL, Mulcahy VL, Crick K, Fear C, Robinson S, Swift L, Gambardella L, Bargehr J, Ortmann D, Brown SE, Osnato A, Murphy MP, Corbett G, Gelson WTH, Mells GF, Humphreys P, Davies SE, Amin I, Gibbs P, Sinha S, Teichmann SA, Butler AJ, See TC, Melum E, Watson CJE, Saeb-Parsy K, and Vallier L**. Cholangiocyte organoids can repair bile ducts after transplantation in the human liver. *Science* 371: 839-846, 2021.
11. **Peters-Hall JR, Coquelin ML, Torres MJ, LaRanger R, Alabi BR, Sho S, Calva-Moreno JF, Thomas PJ, and Shay JW**. Long-term culture and cloning of primary human bronchial basal cells that maintain multipotent differentiation capacity and CFTR channel function. *Am J Physiol Lung Cell Mol Physiol* 315: L313-L327, 2018.
12. **Feranchak AP, Berl T, Capasso J, Wojtaszek PA, Han J, and Fitz JG**. p38 MAP kinase modulates liver cell volume through inhibition of membrane Na+ permeability. *J Clin Invest* 108: 1495-1504, 2001.
13. **Dutta AK, Khimji AK, Kresge C, Bugde A, Dougherty M, Esser V, Ueno Y, Glaser SS, Alpini G, Rockey DC, and Feranchak AP**. Identification and functional characterization of TMEM16A, a Ca2+-activated Cl- channel activated by extracellular nucleotides, in biliary epithelium. *J Biol Chem* 286: 766-776, 2011.
14. **Shcheynikov N, Boggs K, Green A, and Feranchak AP**. Identification of the chloride channel, leucine-rich repeat-containing protein 8, subfamily a (LRRC8A), in mouse cholangiocytes. *Hepatology* 76: 1248-1258, 2022.
15. **Feranchak AP, Fitz JG, and Roman RM**. Volume-sensitive purinergic signaling in human hepatocytes. *J Hepatol* 33: 174-182, 2000.
16. **Taylor AL, Kudlow BA, Marrs KL, Gruenert DC, Guggino WB, and Schwiebert EM**. Bioluminescence detection of ATP release mechanisms in epithelia. *Am J Physiol* 275: C1391-1406, 1998.

17. **Woo K, Sathe M, Kresge C, Esser V, Ueno Y, Venter J, Glaser SS, Alpini G, and Feranchak AP**. Adenosine triphosphate release and purinergic (P2) receptor-mediated secretion in small and large mouse cholangiocytes. *Hepatology* 52: 1819-1828, 2010.
18. **Fiorotto R, Villani A, Kourtidis A, Scirpo R, Amenduni M, Geibel PJ, Cadamuro M, Spirli C, Anastasiadis PZ, and Strazzabosco M**. The cystic fibrosis transmembrane conductance regulator controls biliary epithelial inflammation and permeability by regulating Src tyrosine kinase activity. *Hepatology* 64: 2118-2134, 2016.
19. **Patrick AE, Karamyshev AL, Millen L, and Thomas PJ**. Alteration of CFTR transmembrane span integration by disease-causing mutations. *Mol Biol Cell* 22: 4461-4471, 2011.
20. **Voss FK, Ullrich F, Munch J, Lazarow K, Lutter D, Mah N, Andrade-Navarro MA, von Kries JP, Stauber T, and Jentsch TJ**. Identification of LRRC8 heteromers as an essential component of the volume-regulated anion channel VRAC. *Science* 344: 634-638, 2014.
21. **Li Q, Dutta A, Kresge C, Bugde A, and Feranchak AP**. Bile acids stimulate cholangiocyte fluid secretion by activation of transmembrane member 16A Cl(-) channels. *Hepatology* 68: 187-199, 2018.
22. **Boyer JL**. Bile formation and secretion. *Compr Physiol* 3: 1035-1078, 2013.
23. **Kudira R, Sharma BK, Mullen M, Mohanty SK, Donnelly B, Tiao GM, and Miethke A**. Isolation and Culturing Primary Chaolangiocytes from Mouse Liver. *Bio Protoc* 11: e4192, 2021.
24. **Vroman B, and LaRusso NF**. Development and characterization of polarized primary cultures of rat intrahepatic bile duct epithelial cells. *Lab Invest* 74: 303-313, 1996.
25. **Salter KD, Roman RM, LaRusso NR, Fitz JG, and Doctor RB**. Modified culture conditions enhance expression of differentiated phenotypic properties of normal rat cholangiocytes. *Lab Invest* 80: 1775-1778, 2000.
26. **Lazaridis KN, Pham L, Tietz P, Marinelli RA, deGroen PC, Levine S, Dawson PA, and LaRusso NF**. Rat cholangiocytes absorb bile acids at their apical domain via the ileal sodium-dependent bile acid transporter. *J Clin Invest* 100: 2714-2721, 1997.
27. **Lazaridis KN, Pham L, Vroman B, de Groen PC, and LaRusso NF**. Kinetic and molecular identification of sodium-dependent glucose transporter in normal rat cholangiocytes. *Am J Physiol* 272: G1168-1174, 1997.
28. **Spirli C, Granato A, Zsembery K, Anglani F, Okolicsanyi L, LaRusso NF, Crepaldi G, and Strazzabosco M**. Functional polarity of Na+/H+ and Cl-/HCO3- exchangers in a rat cholangiocyte cell line. *Am J Physiol* 275: G1236-1245, 1998.
29. **Yang L, Faris RA, and Hixson DC**. Long-term culture and characteristics of normal rat liver bile duct epithelial cells. *Gastroenterology* 104: 840-852, 1993.
30. **Daley WP, Peters SB, and Larsen M**. Extracellular matrix dynamics in development and regenerative medicine. *J Cell Sci* 121: 255-264, 2008.
31. **Riento K, and Ridley AJ**. Rocks: multifunctional kinases in cell behaviour. *Nat Rev Mol Cell Biol* 4: 446-456, 2003.
32. **Saadeldin IM, Tukur HA, Aljumaah RS, and Sindi RA**. Rocking the Boat: The Decisive Roles of Rho Kinases During Oocyte, Blastocyst, and Stem Cell Development. *Front Cell Dev Biol* 8: 616762, 2020.
33. **Watanabe K, Ueno M, Kamiya D, Nishiyama A, Matsumura M, Wataya T, Takahashi JB, Nishikawa S, Nishikawa S, Muguruma K, and Sasai Y**. A ROCK inhibitor permits survival of dissociated human embryonic stem cells. *Nat Biotechnol* 25: 681-686, 2007.
34. **Joo HJ, Choi DK, Lim JS, Park JS, Lee SH, Song S, Shin JH, Lim DS, Kim I, Hwang KC, and Koh GY**. ROCK suppression promotes differentiation and expansion of endothelial cells from embryonic stem cell-derived Flk1(+) mesodermal precursor cells. *Blood* 120: 2733-2744, 2012.
35. **Kurosawa H**. Application of Rho-associated protein kinase (ROCK) inhibitor to human pluripotent stem cells. *J Biosci Bioeng* 114: 577-581, 2012.

36. **Chapman S, Liu X, Meyers C, Schlegel R, and McBride AA**. Human keratinocytes are efficiently immortalized by a Rho kinase inhibitor. *J Clin Invest* 120: 2619-2626, 2010.
37. **van den Bogaard EH, Rodijk-Olthuis D, Jansen PA, van Vlijmen-Willems IM, van Erp PE, Joosten I, Zeeuwen PL, and Schalkwijk J**. Rho kinase inhibitor Y-27632 prolongs the life span of adult human keratinocytes, enhances skin equivalent development, and facilitates lentiviral transduction. *Tissue Eng Part A* 18: 1827-1836, 2012.
38. **Dale TP, Borg D'anastasi E, Haris M, and Forsyth NR**. Rock Inhibitor Y-27632 Enables Feeder-Free, Unlimited Expansion of Sus scrofa domesticus Swine Airway Stem Cells to Facilitate Respiratory Research. *Stem Cells Int* 2019: 3010656, 2019.
39. **Jeong D, Han C, Kang I, Park HT, Kim J, Ryu H, Gho YS, and Park J**. Effect of Concentrated Fibroblast-Conditioned Media on In Vitro Maintenance of Rat Primary Hepatocyte. *PLoS One* 11: e0148846, 2016.
40. **Liu X, Ory V, Chapman S, Yuan H, Albanese C, Kallakury B, Timofeeva OA, Nealon C, Dakic A, Simic V, Haddad BR, Rhim JS, Dritschilo A, Riegel A, McBride A, and Schlegel R**. ROCK inhibitor and feeder cells induce the conditional reprogramming of epithelial cells. *Am J Pathol* 180: 599-607, 2012.
41. **Li Z, Han S, Wang X, Han F, Zhu X, Zheng Z, Wang H, Zhou Q, Wang Y, Su L, Shi J, Tang C, and Hu D**. Rho kinase inhibitor Y-27632 promotes the differentiation of human bone marrow mesenchymal stem cells into keratinocyte-like cells in xeno-free conditioned medium. *Stem Cell Res Ther* 6: 17, 2015.
42. **Gentzsch M, Boyles SE, Cheluvaraju C, Chaudhry IG, Quinney NL, Cho C, Dang H, Liu X, Schlegel R, and Randell SH**. Pharmacological Rescue of Conditionally Reprogrammed Cystic Fibrosis Bronchial Epithelial Cells. *Am J Respir Cell Mol Biol* 56: 568-574, 2017.
43. **Francis H, Glaser S, Demorrow S, Gaudio E, Ueno Y, Venter J, Dostal D, Onori P, Franchitto A, Marzioni M, Vaculin S, Vaculin B, Katki K, Stutes M, Savage J, and Alpini G**. Small mouse cholangiocytes proliferate in response to H1 histamine receptor stimulation by activation of the IP3/CaMK I/CREB pathway. *Am J Physiol Cell Physiol* 295: C499-513, 2008.
44. **Tabibian JH, Trussoni CE, O'Hara SP, Splinter PL, Heimbach JK, and LaRusso NF**. Characterization of cultured cholangiocytes isolated from livers of patients with primary sclerosing cholangitis. *Lab Invest* 94: 1126-1133, 2014.

# 8921_0_art_file_212226_t55s55.docx

ORIGINALITY REPORT

# 41%

SIMILARITY INDEX

PRIMARY SOURCES

1 Qin Li, Charles Kresge, Kristy Boggs, Julie Scott, Andrew Feranchak. "Mechanosensor transient receptor potential vanilloid member 4 (TRPV4) regulates mouse cholangiocyte secretion and bile formation", American Journal of Physiology-Gastrointestinal and Liver Physiology, 2020 — 686 words — 8%
Crossref

2 Nikolay Shcheynikov, Kristy Boggs, Anthony Green, Andrew P. Feranchak. "Identification of the Chloride Channel, Leucine-Rich Repeat-Containing Protein 8, subfamily A (LRRC8A), in Mouse Cholangiocytes", Hepatology, 2022 — 322 words — 4%
Crossref

3 www.ncbi.nlm.nih.gov — 296 words — 3%
Internet

4 Li Yang, Ronald A. Faris, Douglas C. Hixson. "Long-term culture and characteristics of normal rat liver bile duct epithelial cells", Gastroenterology, 1993 — 251 words — 3%
Crossref

5 Nikolay Shcheynikov, Kristy Boggs, Anthony Green, Andrew P. Feranchak. "Identification of the chloride channel, leucine-rich repeat-containing protein 8, subfamily a (LRRC8A), in mouse cholangiocytes", Hepatology, 2022 — 158 words — 2%
Crossref

6 www.physiology.org — 152 words — 2%
Internet

7 journals.physiology.org

Internet

137 words — 2%

8 www.jbc.org
Internet

103 words — 1%

9 aasldpubs.onlinelibrary.wiley.com
Internet

90 words — 1%

10 "Modified Culture Conditions Enhance Expression of Differentiated Phenotypic Properties of Normal Rat Cholangiocytes", Laboratory Investigation, 11/2000
Crossref

68 words — 1%

11 www.nature.com
Internet

63 words — 1%

12 Amal K. Dutta, Kristy Boggs, Al-karim Khimji, Yonas Getachew, Youxue Wang, Charles Kresge, Don C. Rockey, Andrew P. Feranchak. "Signaling through the interleukin-4 and interleukin-13 receptor complexes regulates cholangiocyte TMEM16A expression and biliary secretion", American Journal of Physiology-Gastrointestinal and Liver Physiology, 2020
Crossref

59 words — 1%

13 Li, Qin, Charles Kresge, Abhijit Bugde, Michelle Lamphere, Jason Y. Park, and Andrew P. Feranchak. "Regulation of mechanosensitive biliary epithelial transport by the Epithelial Na+ Channel, ENaC : Cholangiocyte ENaC contributes to Na+ transport", Hepatology, 2015.
Crossref

56 words — 1%

14 www.mdpi.com
Internet

51 words — 1%

15 www.biorxiv.org
Internet

50 words — 1%

www.hindawi.com

| | | |
|---|---|---|
| 16 | Internet | 46 words — 1% |
| 17 | Abigail Soyombo. "Gating of CFTR by the STAS domain of SLC26 transporters", Nature Cell Biology, 04/2004<br>Crossref | 43 words — < 1% |
| 18 | www.scinapse.io<br>Internet | 41 words — < 1% |
| 19 | stemcellres.biomedcentral.com<br>Internet | 40 words — < 1% |
| 20 | Mary Ayers, Karis Kosar, Yuhua Xue, Chhavi Goel et al. "Inhibiting Wnt signaling reduces cholestatic injury by disrupting the inflammatory axis", Cellular and Molecular Gastroenterology and Hepatology, 2023<br>Crossref | 36 words — < 1% |
| 21 | wscpr.org<br>Internet | 34 words — < 1% |
| 22 | www.jci.org<br>Internet | 32 words — < 1% |
| 23 | Amal K. Dutta, Al-Karim Khimji, Songling Liu, Zemfira Karamysheva et al. " PKCα regulates TMEM16A-mediated Cl secretion in human biliary cells ", American Journal of Physiology-Gastrointestinal and Liver Physiology, 2016<br>Crossref | 31 words — < 1% |
| 24 | healthdocbox.com<br>Internet | 30 words — < 1% |
| 25 | www.researchsquare.com<br>Internet | 30 words — < 1% |

| | | | |
|---|---|---|---|
| 26 | Sucheta Kulkarni, Qin Li, Aatur D. Singhi, Silvia Liu, Satdarshan P. Monga, Andrew P. Feranchak. "TMEM16A partners with mTOR to influence pathways of cell survival, proliferation, and migration in cholangiocarcinoma", American Journal of Physiology-Gastrointestinal and Liver Physiology, 2023<br>Crossref | 26 words | < 1% |
| 27 | doaj.org<br>Internet | 22 words | < 1% |
| 28 | link.springer.com<br>Internet | 20 words | < 1% |
| 29 | addi.ehu.eus<br>Internet | 19 words | < 1% |
| 30 | d.docksci.com<br>Internet | 18 words | < 1% |
| 31 | escholarship.org<br>Internet | 18 words | < 1% |
| 32 | Lucie Oberhauser, Sabrina Granziera, Adai Colom, Antoine Goujon et al. "Palmitate and oleate modify membrane fluidity and kinase activities of INS-1E β-cells alongside altered metabolism-secretion coupling", Biochimica et Biophysica Acta (BBA) - Molecular Cell Research, 2020<br>Crossref | 17 words | < 1% |
| 33 | pubmed.ncbi.nlm.nih.gov<br>Internet | 17 words | < 1% |
| 34 | df6sxcketz7bb.cloudfront.net<br>Internet | 16 words | < 1% |
| 35 | dokumen.pub<br>Internet | 16 words | < 1% |

| | | |
|---|---|---|
| 36 | Kari Nejak-Bowen. "If It Looks Like a Duct and Acts Like a Duct: On the Role of Reprogrammed Hepatocytes in Cholangiopathies", Gene Expression, 2020<br>Crossref | 15 words — < 1% |
| 37 | academic.oup.com<br>Internet | 15 words — < 1% |
| 38 | Dianat, Noushin, Hélène Dubois-Pot-Schneider, Clara Steichen, Christophe Desterke, Philippe Leclerc, Aurélien Raveux, Laurent Combettes, Anne Weber, Anne Corlu, and Anne Dubart-Kupperschmitt. "Generation of functional cholangiocyte-like cells from human pluripotent stem cells and HepaRG cells", Hepatology, 2014.<br>Crossref | 14 words — < 1% |
| 39 | Tadashi Yamamoto, Yuki Ugawa, Mari Kawamura, Keisuke Yamashiro, Shinsuke Kochi, Hidetaka Ideguchi, Shogo Takashiba. "Modulation of microenvironment for controlling the fate of periodontal ligament cells: the role of Rho/ROCK signaling and cytoskeletal dynamics", Journal of Cell Communication and Signaling, 2017<br>Crossref | 14 words — < 1% |
| 40 | dspace.uohyd.ac.in<br>Internet | 14 words — < 1% |
| 41 | Casey A. Rimland, Samantha G. Tilson, Carola M. Morell, Rute A. Tomaz et al. " Regional Differences in Human Biliary Tissues and Corresponding – Derived Organoids ", Hepatology, 2021<br>Crossref | 13 words — < 1% |
| 42 | Jennifer R. Peters-Hall, Melissa L. Coquelin, Michael J. Torres, Ryan LaRanger et al. "Long-term culture and cloning of primary human bronchial basal cells that maintain multipotent differentiation capacity and CFTR channel | 13 words — < 1% |

function", American Journal of Physiology-Lung Cellular and Molecular Physiology, 2018
Crossref

43 Seitaro Komai, Mayumi Ueta, Hiromi Nishigaki, Katsura Mizushima, Yuji Naito, Shigeru Kinoshita, Chie Sotozono. "Differences of gene regulation by TLR3 and IPS-1 in polyI:C-stimulated murine corneal epithelial cells", Research Square Platform LLC, 2022 — 13 words — < 1%
Crossref Posted Content

44 ajpgi.physiology.org — 13 words — < 1%
Internet

45 jpet.aspetjournals.org — 13 words — < 1%
Internet

46 Angela C. Cheung, Maria J. Lorenzo Pisarello, Nicholas F. LaRusso. "Pathobiology of biliary epithelia", Biochimica et Biophysica Acta (BBA) - Molecular Basis of Disease, 2018 — 12 words — < 1%
Crossref

47 Zhenzhen Li, Shichao Han, Xingqin Wang, Fu Han, Xiongxiang Zhu, Zhao Zheng, Hongtao Wang, Qin Zhou, Yunchuan Wang, Linlin Su, Jihong Shi, Chaowu Tang, Dahai Hu. "Rho kinase inhibitor Y-27632 promotes the differentiation of human bone marrow mesenchymal stem cells into keratinocyte-like cells in xeno-free conditioned medium", Stem Cell Research & Therapy, 2015 — 12 words — < 1%
Crossref

48 www.microvesicles.org — 12 words — < 1%
Internet

49 Claudio Pinto, Debora Maria Giordano, Luca Maroni, Marco Marzioni. "Role of inflammation and proinflammatory cytokines in cholangiocyte — 11 words — < 1%

pathophysiology", Biochimica et Biophysica Acta (BBA) - Molecular Basis of Disease, 2018
Crossref

50 journals.lww.com — 11 words — < 1%
Internet

51 Amal K. Dutta, Kangmee Woo, Al-karim Khimji, Charles Kresge et al. "Mechanosensitive Cl −", 'American Physiological Society' — 10 words — < 1%
Internet

52 Hasina Akhter, Wen-Tan Huang, Thomas van Groen, Hui-Chien Kuo, Toshio Miyata, Rui-Ming Liu. "A Small Molecule Inhibitor of Plasminogen Activator Inhibitor-1 Reduces Brain Amyloid-β Load and Improves Memory in an Animal Model of Alzheimer's Disease", Journal of Alzheimer's Disease, 2018 — 10 words — < 1%
Crossref

53 Jie Fu, ZhaoBing Gao, Bing Shen, Michael X. Zhu. "Canonical transient receptor potential 4 and its small molecule modulators", Science China Life Sciences, 2014 — 10 words — < 1%
Crossref

54 "Calcium transport study of SF-9 lepidopteran cells and bull frog sympathetic ganglion cells", University/Medicine and Dentistry/Medicine/Anatomy & Physiology, 2006-06-20 — 9 words — < 1%
Publications

55 Brendon M Baker, Andrew M Handorf, Lara C Ionescu, Wan-Ju Li, Robert L Mauck. "New directions in nanofibrous scaffolds for soft tissue engineering and regeneration", Expert Review of Medical Devices, 2014 — 9 words — < 1%
Crossref

56 journals.biologists.com — 9 words — < 1%
Internet

57 krummellab.com
Internet
9 words — < 1%

58 www.exocarta.org
Internet
9 words — < 1%

59 www.spandidos-publications.com
Internet
9 words — < 1%

60 Ewa Krawczyk. "Conditionally Reprogrammed Cells as Preclinical Model for Rare Cancers", Cancers, 2025
Crossref
8 words — < 1%

61 Manju R Purohit, Tehmina Mustafa, Harald G Wiker, Odd Mørkve, Lisbet Sviland. "Immunohistochemical diagnosis of abdominal and lymph node tuberculosis by detecting Mycobacterium tuberculosis complex specific antigen MPT64", Diagnostic Pathology, 2007
Crossref
8 words — < 1%

62 Molecular Pathology Library, 2011.
Crossref
8 words — < 1%

63 Wei Liu, Sheng-Ning Zhang, Zong-Qiang Hu, Shi-Ming Feng, Zhen-Hui Li, Shu-Feng Xiao, Hong-Shu Wang, Li Li. "Study of Recellularized Human Acellular Arterial Matrix Repairs Porcine Biliary Segmental Defects", Tissue Engineering and Regenerative Medicine, 2019
Crossref
8 words — < 1%

64 Wencheng Zhang, Yangyang Cui, Yuan Du, Yong Yang et al. "Liver cell therapies: cellular sources and grafting strategies", Frontiers of Medicine, 2023
Crossref
8 words — < 1%

65 Xiafang Xu, Xionghua Sun, Xuelei Wan, Xihua Chen, Xiaogang Jiang. "Mitomycin induces alveolar
8 words — < 1%

epithelial cell senescence by down-regulating GSK3β signaling", Toxicology Letters, 2021
Crossref

66 Yi Xiao, Hao Zhang, Xu Liu, Pengfei Xu et al. "Medium from human iPSC-derived primitive macrophages promotes adult cardiomyocyte proliferation and cardiac regeneration", Nature Communications, 2025 — 8 words — < 1%
Crossref

67 ajpcell.physiology.org — 8 words — < 1%
Internet

68 coek.info — 8 words — < 1%
Internet

69 microvesicles.org — 8 words — < 1%
Internet

70 pmc.ncbi.nlm.nih.gov — 8 words — < 1%
Internet

71 serval.unil.ch — 8 words — < 1%
Internet

72 www.bioinfor.com — 8 words — < 1%
Internet

73 www.science.gov — 8 words — < 1%
Internet

74 "Ion Channels and Transporters of Epithelia in Health and Disease", Springer Nature, 2016 — 7 words — < 1%
Crossref

75 Meghana N. Sathe, Kangmee Woo, Charles Kresge, Abhijit Bugde, Kate Luby-Phelps, Matthew A. Lewis, Andrew P. Feranchak. "Regulation of Purinergic Signaling in Biliary Epithelial Cells by Exocytosis of SLC17A9-dependent ATP-enriched Vesicles", Journal of Biological Chemistry, 2011 — 7 words — < 1%

Crossref

76 Nikhil T. Awatade, Sharon L. Wong, Chris K. Hewson, Laura K. Fawcett, Anthony Kicic, Adam Jaffe, Shafagh A. Waters. "Human Primary Epithelial Cell Models: Promising Tools in the Era of Cystic Fibrosis Personalized Medicine", Frontiers in Pharmacology, 2018 — 7 words — < 1%
Crossref

77 Qin Li, Amal Dutta, Charles Kresge, Abhijit Bugde, Andrew P. Feranchak. " Bile acids stimulate cholangiocyte fluid secretion by activation of transmembrane member 16A Cl channels ", Hepatology, 2018 — 7 words — < 1%
Crossref

78 Signaling Pathways in Liver Diseases, 2005. — 7 words — < 1%
Crossref

79 "Hepatocytes", Springer Science and Business Media LLC, 2022 — 6 words — < 1%
Crossref

80 A. P. Feranchak. "p38 MAP kinase modulates liver cell volume through inhibition of membrane Na+ permeability", Journal of Clinical Investigation, 11/15/2001 — 6 words — < 1%
Crossref

81 Amal K. Dutta, Kangmee Woo, R. Brian Doctor, J. Gregory Fitz, Andrew P. Feranchak. " Extracellular nucleotides stimulate Cl currents in biliary epithelia through receptor-mediated IP3 and Ca release ", American Journal of Physiology-Gastrointestinal and Liver Physiology, 2008 — 6 words — < 1%
Crossref

82 Boning Gao, Chunxian Huang, Kemp Kernstine, Vasiliki Pelekanou et al. "Non-malignant respiratory epithelial cells preferentially proliferate from resected non-small cell lung cancer specimens cultured under conditionally reprogrammed conditions", Oncotarget, 2016 — 6 words — < 1%
Crossref

83 James J. Matsuda, Mohammed S. Filali, Kenneth A. Volk, Malia M. Collins, Jessica G. Moreland, Fred S. Lamb. "Overexpression of CLC-3 in HEK293T cells yields novel currents that are pH dependent", American Journal of Physiology-Cell Physiology, 2008
Crossref
6 words — < 1%

84 Kangmee Woo, Meghana Sathe, Charles Kresge, Victoria Esser et al. "Adenosine Triphosphate Release and Purinergic (P2) Receptor–Mediated Secretion in Small and Large Mouse Cholangiocytes", Hepatology, 2010
Crossref
6 words — < 1%

85 Laura M. Molina, Junjie Zhu, Qin Li, Tirthadipa Pradhan-Sundd et al. "Compensatory hepatic adaptation accompanies permanent absence of intrahepatic biliary network due to YAP1 loss in liver progenitors", Cell Reports, 2021
Crossref
6 words — < 1%

86 Miguel Prudêncio. "<i>Plasmodium berghei</i>-infection induces volume-regulated anion channel-like activity in human hepatoma cells", Cellular Microbiology, 10/2009
Crossref
6 words — < 1%

87 Ravshan Z. Sabirov, Amal K. Dutta, Yasunobu Okada. "Volume-Dependent Atp-Conductive Large-Conductance Anion Channel as a Pathway for Swelling-Induced Atp Release", The Journal of General Physiology, 2001
Crossref
6 words — < 1%

EXCLUDE QUOTES OFF
EXCLUDE BIBLIOGRAPHY ON
EXCLUDE SOURCES OFF
EXCLUDE MATCHES OFF
